# The Impact of Growth Hormone Therapy on the Apoptosis Assessment in CD34+ Hematopoietic Cells from Children with Growth Hormone Deficiency

**DOI:** 10.3390/ijms18010111

**Published:** 2017-01-07

**Authors:** Miłosz Piotr Kawa, Iwona Stecewicz, Katarzyna Piecyk, Edyta Paczkowska, Dorota Rogińska, Anna Sobuś, Karolina Łuczkowska, Ewa Pius-Sadowska, Elżbieta Gawrych, Elżbieta Petriczko, Mieczysław Walczak, Bogusław Machaliński

**Affiliations:** 1 Department of General Pathology, Pomeranian Medical University in Szczecin, 72 Powstancow Wlkp. Street, 70-111 Szczecin, Poland; kawamilosz@gmail.com (M.P.K.); ka.kaczynska@gmail.com (K.P.); edyta.paczkowska@pum.edu.pl (E.P.); doroginska@gmail.com (D.R.); ania.sobus@gmail.com (A.S.); karolinaluczkowska58@gmail.com (K.Ł.); ewapius@wp.pl (E.P.-S.); 2 Department of Pediatrics, Endocrinology, Diabetology, Metabolic Diseases and Cardiology of the Developmental Age, Pomeranian Medical University in Szczecin, 1 Unii Lubelskiej Street, 71-252 Szczecin, Poland; iwona.stecewicz@wp.pl (I.S.); petrela1@wp.pl (E.P.); klchrdz2@pum.edu.pl (M.W.); 3 Department of Pediatric and Oncological Surgery, Pomeranian Medical University in Szczecin, 1 Unii Lubelskiej Street, 71-252 Szczecin, Poland; chirurgia.dziecieca@op.pl

**Keywords:** CD34+ cells, hematopoietic progenitor cells, hematopoiesis, growth hormone deficiency, growth hormone deficiency in children, apoptosis, cell death, apoptosis control

## Abstract

Growth hormone (GH) modulates hematopoietic cell homeostasis and is associated with apoptosis control, but with limited mechanistic insights. Aim of the study was to determine whether GH therapeutic supplementation (GH-TS) could affect apoptosis of CD34+ cells enriched in hematopoietic progenitor cells of GH deficient (GHD) children. CD34+ cells from peripheral blood of 40 GHD children were collected before and in 3rd and 6th month of GH-TS and compared to 60 controls adjusted for bone age, sex, and pubertal development. Next, apoptosis assessment via different molecular techniques was performed. Finally, to comprehensively characterize apoptosis process, global gene expression profile was determined using genome-wide RNA microarray technology. Results showed that GH-TS significantly reduced spontaneous apoptosis in CD34+ cells (*p* < 0.01) and results obtained using different methods to detect early and late apoptosis in analyzed cells population were consistent. GH-TS was also associated with significant downregulation of several members of TNF-alpha superfamily and other genes associated with apoptosis and stress response. Moreover, the significant overexpression of cyto-protective and cell cycle-associated genes was detected. These findings suggest that recombinant human GH has a direct anti-apoptotic activity in hematopoietic CD34+ cells derived from GHD subjects in course of GH-TS.

## 1. Introduction

Growth hormone (GH) is essential for body growth during childhood and continues to stimulate anabolic processes in adults. GH exerts its anabolic effects largely indirectly via stimulation of insulin-like growth factor-1 (IGF1) production. Components of the GH–IGF1 axis make an important contribution to the development, function, and proliferation of different tissues [1]. Moreover, acting in endocrine/paracrine/autocrine manner, GH binds to the specific GH receptor (GHR), which may induce two downstream pathways: (i) the JAK2-STAT5 pathway that in turn leads to up-regulation of GH responsive elements; and (ii) the RAS/MAPK/ERK pathway leading to enhanced transcriptional activity of GH-regulated genes [1]. The classical examples of IGF1-independent actions mediated through GHR are the proliferation of chondrocyte stem cells at bone growth plate [2], the fusion of myoblasts with nascent myotubes to increase muscle fiber size [3] or direct stimulation of neural stem cells to proliferate [4]. Additionally, GHR has been shown to be expressed in different mature hematopoietic cells [5].

Recent studies have also focused on the anti-apoptotic effects of GH. Apoptosis is the most common, gene-directed form of programmed cell death that contributes to different physiologic and pathologic processes. Apoptosis also plays a key role in controlling the homeostasis of the hematopoietic system. Several lines of evidence indicate that different hormones play a role as regulators of eukaryotic cell death [6]. Likewise, several studies revealed diverse biological actions of GH in different types of cells, including prevention of apoptosis in mature hematopoietic and immune-related cells. For instance, GH has been shown to prevent apoptosis of immune-related cells through different molecular mechanisms, such as down-regulation of pro-apoptotic Fas Ligand in human neutrophils [7], rising the expression of the anti-apoptotic Bcl-2 in primary monocytes, promonocytic cell line [8,9] and human lymphocytes [10] or inhibition of caspase-3 activity in human T and B lymphocyte cell lines [8,9]. On the other hand, GH has been shown to inhibit the production of pro-inflammatory cytokines in many cells, shifting the balance in favor of anti-inflammatory cytokines, which may potentially play an important role in prevention of apoptosis [11]. Importantly, the anti-apoptotic GH effects have also been observed in conditions characterized by increased local GH production. Arnold and Weigent found that GH-overexpressing T cell lymphoma can reduce chemically-induced apoptosis via increased expression of BCL-2, with concurrent decrease in expression of pro-apoptotic molecules [12]. In contrast, the same cells depleted of GH were characterized by the increased apoptosis rate. These results strongly indicate that GH plays an important role in the survival of hematopoietic cells exposed to stressful stimuli. However, most of the studies on the apoptotic activity in selected types of hematopoietic cells are based on in vitro conditions. Data about alterations of programmed cell death process following GH administration in hematopoietic system in vivo are very scarce. Decker et al. observed that the GH treatment of elderly subjects undergoing vascular repair considerably reduced apoptosis in circulating neutrophils in vivo [13].

Patients with GH deficiency (GHD) can display abnormalities in hematopoietic cells circulating in their peripheral blood (PB), including decreased red blood cell mass and quantity [14,15]. Especially, GHD patients frequently suffer from certain types of anemia, including “normocytic-normochromic” anemia [15,16]. The number of white blood cells in GHD patients is frequently decreased too [17]. These findings may be related in part to apoptosis activity in GHD. It was observed that physiological and biochemical abnormalities found in GHD may be modified by GH therapeutic supplementation (GH-TS) [18]. However, data regarding the molecular effects of GH on modulation of cellular processes in hematopoietic progenitor cells are very limited and the biological activities of GH supplementation in these cells need further studies.

In the present study, we investigated the in vivo effects of GH-TS on apoptosis in CD34+ cells enriched in hematopoietic progenitor cells (HPCs) collected from children with isolated GHD. CD34+ cells were examined by different methods for apoptosis activity, including Annexin-V labeling, terminal deoxynucleotidyl transferase-mediated dUTP labeling (TUNEL), and analysis of the expression of apoptosis-related molecules, i.e., BCL-2, BCL-xL, BAX and Caspase-3. Finally, the global gene expression profiles in collected cells were analyzed using RNA microarrays.

## 2. Results

### 2.1. Characteristics of the Clinical Parameters

The characteristics of the subjects enrolled in the study are summarized in Table 1. In total, 100 subjects were evaluated. There were no significant differences regarding the baseline clinical characteristics between the two groups, except with respect to the GH and IGF-1 concentration in peripheral blood. The GH concentration in the GHD patient group after the stimulation tests was markedly lower than normal GH values. In addition, the comparison of levels of IGF-1 in GHD patients revealed that blood levels of IGF-1 were significantly higher (up to 2 times, *p* < 0.001) in the 3rd and 6th month of GH-TS compared to GHD patients before therapy (229.5 and 214.3 vs. 125.0 ng/mL, respectively). Additionally, GHD patients with GH-TS presented significantly higher levels (*p* < 0.05) of IGF-1 than healthy controls (229.5 and 214.3 vs. 162.2 ng/mL, respectively). In contrast, IGF-1 concentration was significantly lower (*p* < 0.05) in GHD patients before therapy than in controls (125.0 vs. 162.2 ng/mL, respectively). We observed no significant differences in IGF-1 levels between both groups of GHD patients with GH-TS treated for 3 and 6 months.

### 2.2. GHR Is Expressed at the Protein Level in CD34+ Hematopoietic Cells from GHD Children

**Figure 1:**
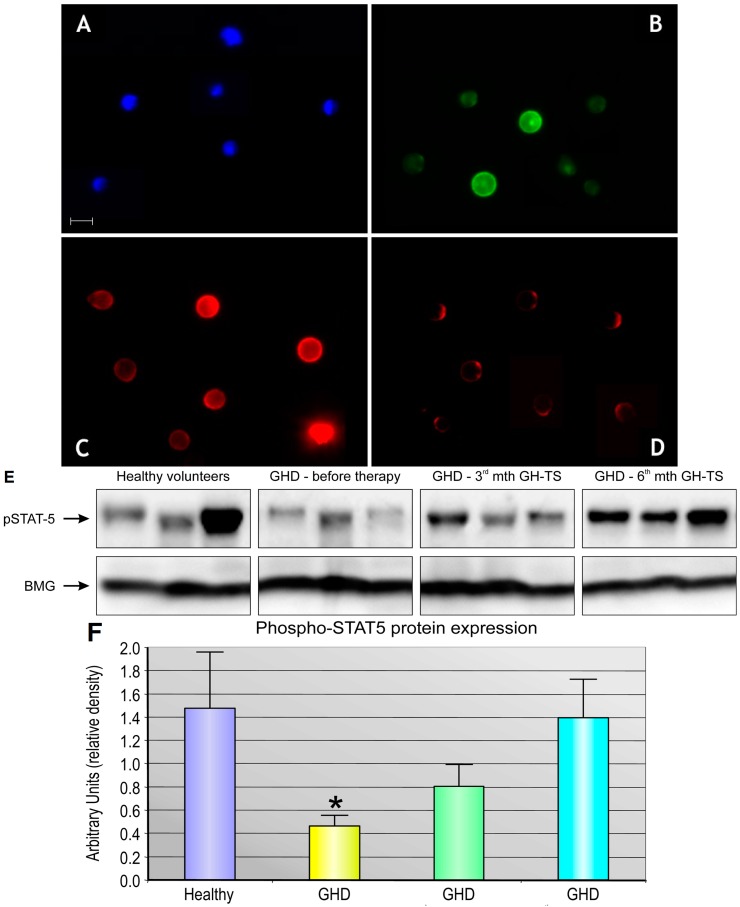
GHR expression in CD34+ cells from GHD patients. The expression of GHR was assessed by immunocytofluorescence in CD34+ cells stained with monoclonal anti-CD45-FITC (**B**) and anti-GHR-PE antibodies (**C**,**D**); The cell nuclei were stained with DAPI (**A**). Cells were harvested from PB of GHD patients before GH-TS (**A**,**B**,**D**) and from their healthy controls (**C**). The expression of each antigen was examined in CD34+ cells of five representative subjects from each group. Representative and selected data are presented. All cells were captured with ×40 objective magnification. Scale bar: 10 µm; The western blot analysis (**E**) and densitometry measurement (**F**) for relative protein quantification of the active, phosphorylated form of STAT-5 (p-STAT) revealed its significantly decreased expression in CD34+ cells from untreated GHD patients and its normal expression in GH-treated GHD patients relative to controls. The band of beta-2-microglobulin (BMG) expression was used as an internal control. Representative and selected data are presented. * *p* < 0.05.

To detect GHR surface protein expression on CD34+ cells, the immunofluorescence (IF) analysis was performed. CD34+ cells from untreated GHD patients and healthy controls expressed GHR protein as shown by positive IF staining exhibited in Figure 1. Interestingly, we observed that GHR immunofluorescence level was slightly decreased in GHD patients compared to their healthy controls. The hematopoietic origin of isolated CD34+ cells was confirmed by detection of surface expression of particular hematopoiesis-related antigen, CD45 (Figure 1B). Subsequently, to confirm whether GH supplementation can induce biological activity of CD34+ cells from GHD patients through GHR, we tested activation of JAK/STAT-signaling pathway in these cells. Therefore, cellular extracts were analyzed by Western blot using antibody that recognizes phosphorylated form of STAT-5. As shown in Figure 1E, we observed stable expression of phopho-STAT-5 protein in CD34+ cells from GHD patients treated with GH-TS, which was not significantly different from the control group. Importantly, in CD34+ cells from untreated GHD patients the expression of phopho-STAT-5 was significantly decreased compared to controls (*p* < 0.05). These results demonstrate that GHRs expressed on CD34+ cells are biologically active and can induce the intracellular signal transduction pathways through binding of the exogenous GH in the course of GH therapy in vivo.

### 2.3. GHR Protein Expression in Individual CD34+ Hematopoietic Cells Is Decreased in GHD Children and Not Changing in the Course of GH Therapy

**Figure 2:**
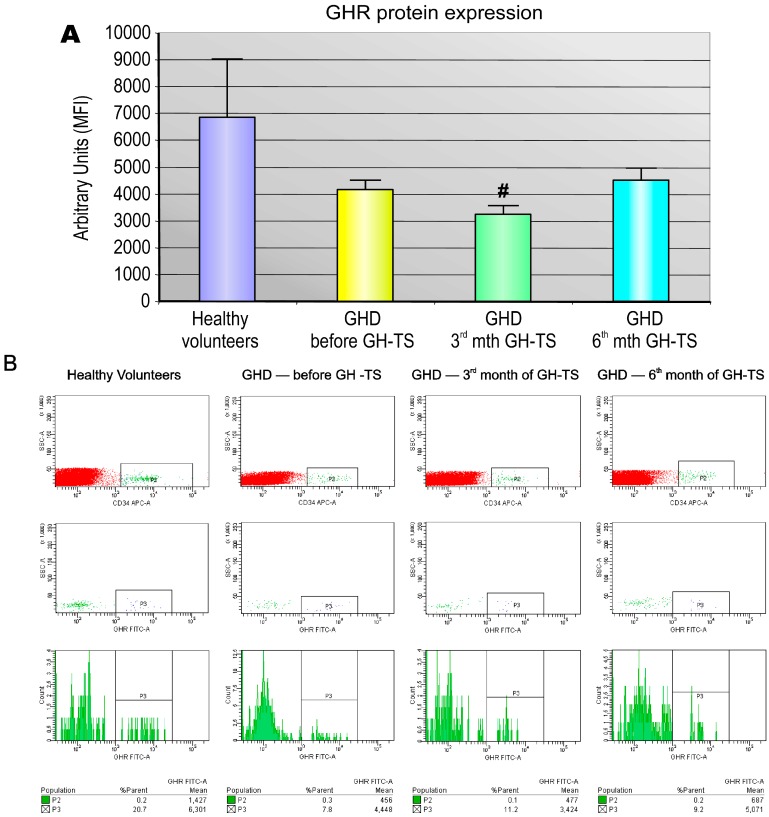
Quantitative analysis of GHR protein density on CD34+ cells from GHD patients. The quantitative analysis of the expression of GHR receptors on the cell membrane surface of CD34+ cells from GHD patients in course of GH therapy was performed (**A**). CD34+ cells were collected from PB of from healthy controls and GHD patients at different time points (before GH-TS and in the 3rd and 6th month of GH-TS). Surface GHR protein expression was assessed by flow cytometric analysis based on the mean fluorescence intensity (MFI) of GHR staining of CD34+ cells. The results are expressed as the mean value ± S.D. # *p* < 0.05 vs. control group. Representative flow cytometric immunofluorescence histograms of GHR expression by CD34+ cells harvested from PB of healthy controls and GHD patients before GH-TS and in the 3rd and 6th month of GH-TS are presented (**B**).

The analysis of the in vivo effects of GH deficiency and its therapeutic supplementation on expression of GHR protein in circulating CD34+ cells was performed using a quantitative cytometric assay to assess the MFI of GHR staining, which corresponds to the concentration of this particular protein on surface of analyzed individual cells (Figure 2). The measured MFI for GHR was significantly decreased in GHD patients after 3 months of GH-TS compared to controls.

### 2.4. GH Therapy Inhibits Apoptosis in CD34+ Hematopoietic Cells from GHD Children

**Figure 3:**
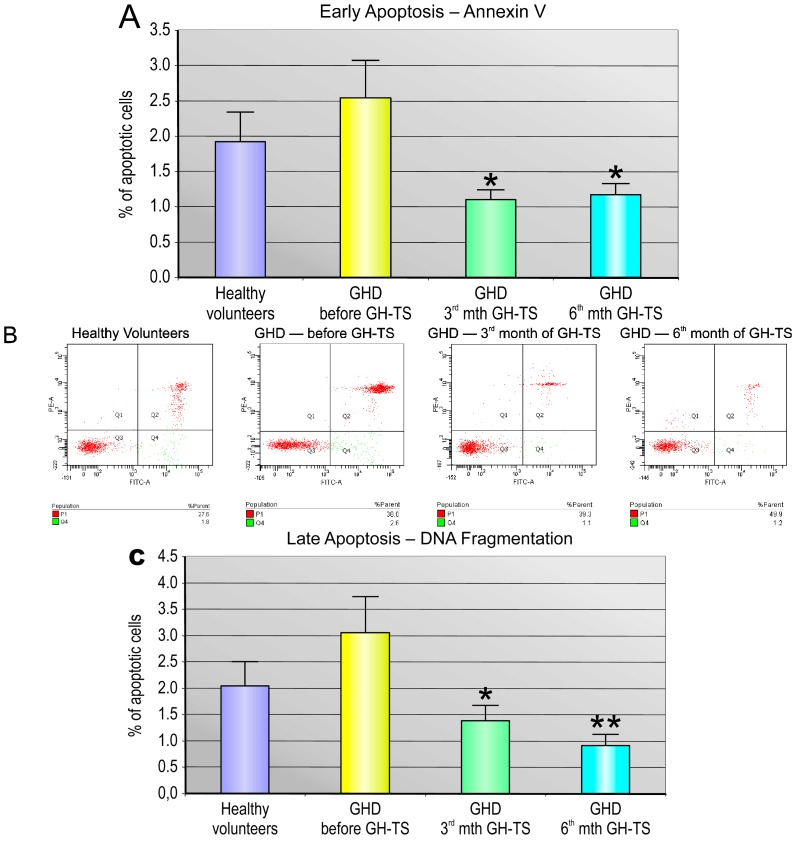

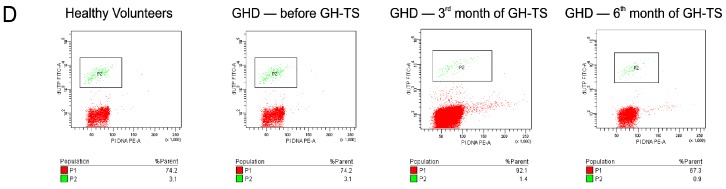
Detection of early and late phase of apoptosis in CD34+ cells from GHD patients. The percentage of CD34+ cells in the early (**A**) and late (**C**) phase of apoptosis was evaluated in control subjects and GHD patients at different time points (before GH-TS, and in the 3rd and 6th months of GH-TS). The results are expressed as the mean value ± S.D. * *p* < 0.05; ** *p* < 0.01 vs. GHD patients prior to GH therapy; Representative flow cytometric quadrant plots for the Annexin V/PI (**B**) and dot plots for the DNA fragmentation (**D**) analysis in CD34+ cells harvested from PB of healthy controls and GHD patients before GH-TS and in the 3rd and 6th month of GH-TS are shown.

To assess the potential in vivo influence of GH on apoptosis in CD34+ cells, two different methods were employed: (i) analysis of the early apoptotic stage characterized by Annexin V, which binds to phosphatidylserine on the cell surface; and (ii) the TUNEL assay, which specifically detects in cell nuclei the fragments of damaged DNA as a result of the late apoptotic stage. Annexin V staining revealed a significant decrease in the percentage of cells undergoing the early apoptosis phase in GHD patients analyzed in the 3rd and 6th month of GH-TS compared to GHD patients before therapy and their controls (Figure 3A). We confirmed these data by analyzing the DNA fragmentation intensity, which demonstrated a significant decrease in the percentage of cells undergoing the late apoptosis phase in GH-treated patients (Figure 3B). Of note, the detected reduction of DNA fragmentation at the later time-point was even more significant than at earlier time-point of GH-TS. Interestingly, there were no significant differences between GHD patients before therapy and their controls (Figure 3A,B). These findings may indicate that supplementation of exogenous GH is involved in the active inhibition of apoptosis in CD34+ cells from GHD children.

### 2.5. GH Therapy Modulates the Gene Expression of Selected Apoptosis-Regulating Proteins in CD34+ Hematopoietic Cells from GHD Children

**Figure 4:**
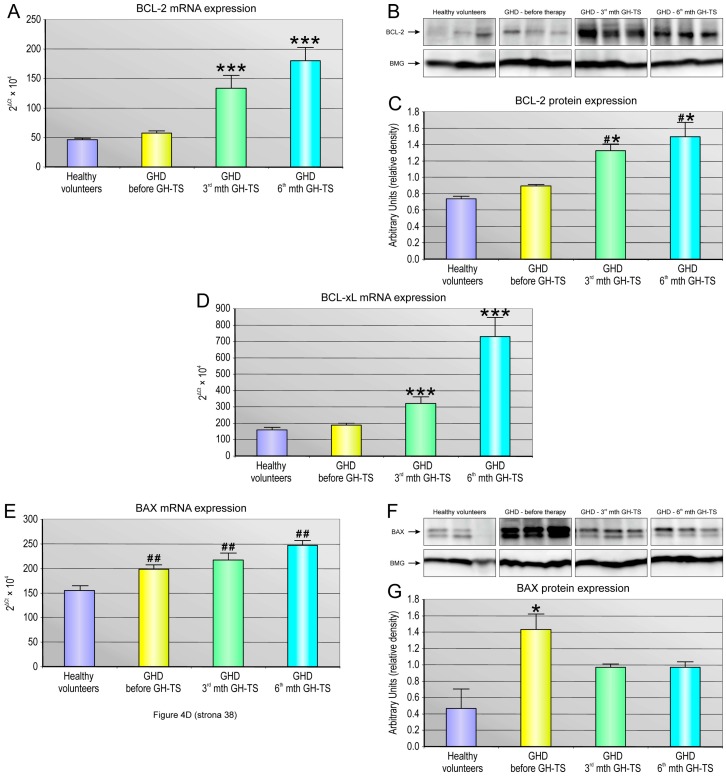

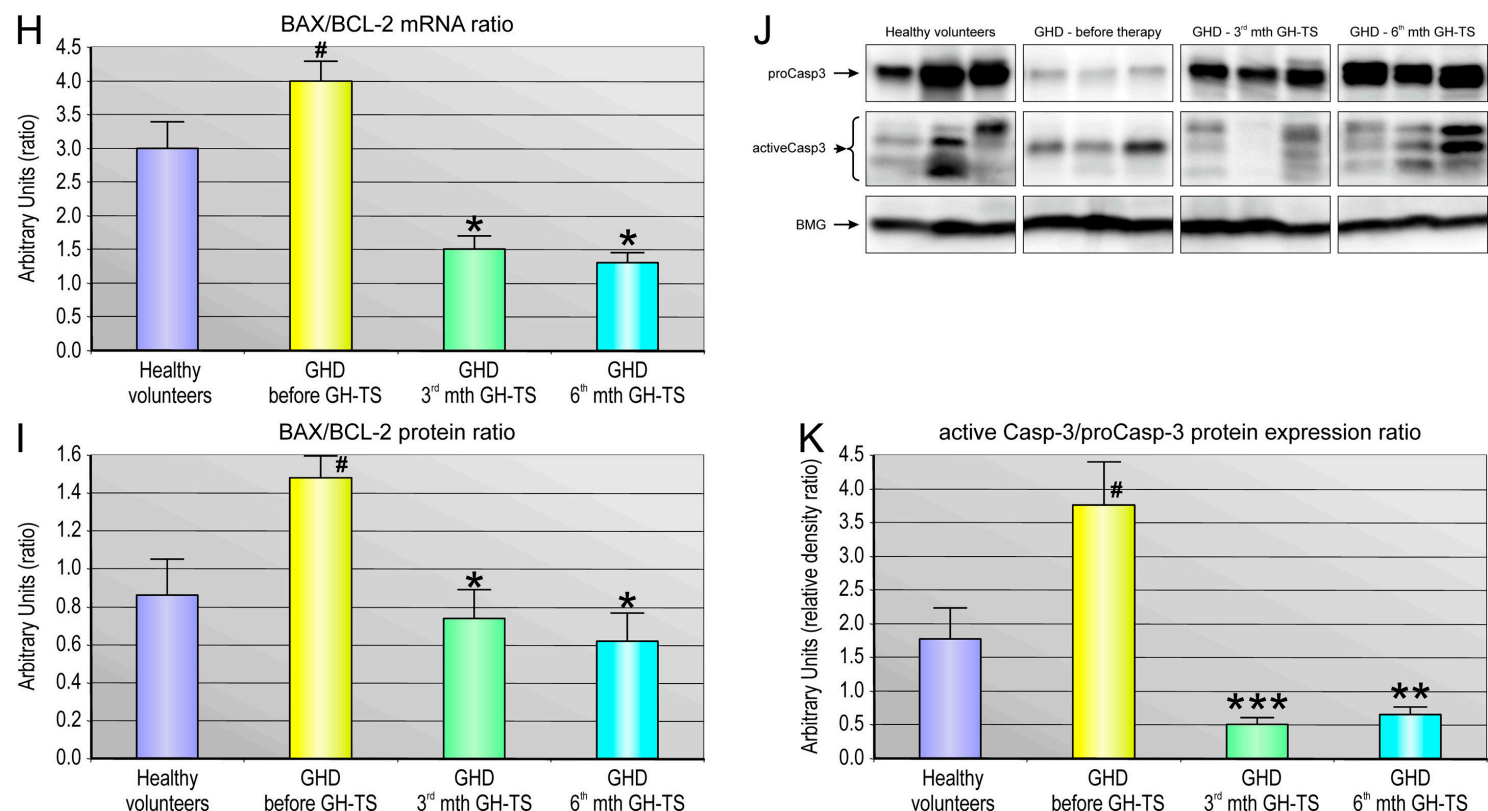
The expression profile of selected apoptosis-related molecules, BCL-2, BCL-XL, BAX and caspase-3 in CD34+ cells from GHD patients. The expression of selected apoptosis-related gene transcripts and proteins was evaluated in CD34+ cells of controls and GHD patients at different time points (before GH-TS and in the 3rd and 6th month of GH-TS). The mRNA expression of BCL-2 (**A**); BCL-XL (**D**), and BAX (**E**) was determined by the quantitative PCR. The mRNA levels are expressed in arbitrary units as the mean value ± S.D. The protein expression of BCL-2 (**B**,**C**); BAX (**F**,**G**); caspase-3 and procaspase-3 (**J**,**K**) was determined by western blot. Beta-2-microglobulin (BMG) served as loading control. Protein bands were analyzed by densitometry. Bars represent mean ± S.D. of selected protein to BMG ratio calculated in all examined groups. The ratio between the optical densities measured in 11-, 17- and 20-kDa cleaved caspase-3 and 32-kDa procaspase-3 bands was calculated as an index of caspase-3 activation. In addition, the BAX/BCL-2 mRNA (**H**) and protein (**I**) ratio was calculated. * *p* < 0.05 vs. GHD patients prior to GH-TS. ** *p* < 0.01 vs. GHD patients prior to GH-TS. *** *p* < 0.001 vs. GHD patients prior to GH-TS. # *p* < 0.05 vs. control values. ## *p* < 0.01 vs. control values.

Next, to analyze the expression levels of anti- and pro-apoptotic genes in CD34+ cells collected from all subjects enrolled to the study, we performed a molecular analysis of mRNA expression for two important anti-apoptotic genes, BCL-2 and BCL-xL, and the main pro-apoptotic BAX gene. Quantitative analysis revealed significantly increased anti-apoptotic gene expression in GHD children after 3 and 6 months of GH-TS compared to GHD patients before therapy and their controls. As shown in Figure 4, we observed a significant 200% up-regulation of BCL-2 mRNA (*p* < 0.001; Figure 4A) and a nearly 100% increase in the expression of BCL-XL mRNA (*p* < 0.001; Figure 4C) in patients treated with GH for 3 months. Moreover, we observed even more pronounced increases in the expression of mRNA for both anti-apoptotic genes in patients treated with GH for 6 months (nearly 300% up-regulation of BCL-2 and BCL-XL mRNA; *p* < 0.001; Figure 4A,C). To further confirm obtained results, we performed a western blot analysis of CD34+ cells collected in all experimental time points using anti-BCL-2 antibody. As shown in Figure 4B, we observed the significantly increased expression of BCL-2 protein in GH-treated patients compared to GHD subjects and healthy controls. Importantly, the CD34+ cells from all analyzed GHD patients (before and during GH-TS) showed a significant increase in the mRNA expression of pro-apoptotic BAX compared to controls (*p* < 0.01; Figure 4D). Likewise, we confirmed obtained results in western blot analysis, and the expression of BAX protein was significantly increased in the GHD patients (*p* < 0.001) and it was also over-expressed in the 3rd and 6th month of the GH-TS (Figure 4E). Overall, these findings indicate the increase in the relative expression of selected anti-apoptotic genes during the six-month-period of GH-TS. Nevertheless, the increase in the expression of pro-apoptotic BAX at gene and protein level in all GHD children was also seen. Apoptosis is tightly controlled by the balance between pro- and anti-apoptotic members of the BCL-2 protein family. Changes in the relative expression levels of such molecules will ultimately decide the cell fate. Therefore, we subsequently evaluated the ratio of transcript and protein levels of pro-apoptotic BAX and anti-apoptotic BCL-2 molecules that defines cell propensity to apoptosis. Importantly, the BAX/BCL-2 mRNA ratio was clearly higher before GH therapy and decreased gradually within the course of GH-TS in a significant mode (Figure 4F). Similarly, the BAX/BCL-2 protein ratio was significantly greater before GH therapy and decreased gradually within the therapy (Figure 4G). These findings suggest that GH is strongly involved in modulation of the expression of selected genes encoding apoptosis-regulating proteins. To further confirm our results, we used western blot analysis to compare the caspase-3 activation in CD34+ cells from GHD patients in course of GH therapy. The cleaved caspase-3/procaspase-3 ratio is considered as an index of caspase-3 activation. As shown in Figure 4H, the ratio between the active form of caspase-3 and its inactive form was significantly higher in GHD patients compared to controls (*p* < 0.05). This finding might indicate that GH deficiency triggers intracellular mechanisms that are essential for apoptosis induction. Moreover, we found that the GH treatment significantly decreased the cleaved caspase-3/procaspase-3 expression ratio in 3rd month of GH-TS, compared to untreated GHD patients (*p* < 0.001) and it continued in a sustained fashion until 6th month of GH-TS (*p* < 0.01). These results would indicate that GH treatment may potentially reduce the executive apoptotic phase in CD34+ cells of GHD patients.

### 2.6. Microarray Analysis of Apoptosis-Related Gene Expression Changes in CD34+ Hematopoietic Cells over 6 Months of GH Treatment

To analyze the mechanism(s) underlying the regulation of apoptosis process by exogenous GH, total RNA was isolated from CD34+ cells collected from controls and GHD patients at different time points (i.e., before GH-TS, and after 6 months of GH-TS), and it was subjected to analysis using RNA microarrays containing approximately 19,900 mRNA transcripts. Next, the genes that showed statistically significant levels of differential regulation by GH (*p* < 0.05) were specifically clustered within functional groups involved in apoptosis control, i.e., related to apoptosis progression (“pro-apoptotic” genes) or its inhibition (“anti-apoptotic” genes). In the former group, we discovered that 33 genes were differentially regulated (Table 2), of which the majority (25 genes) were down-regulated (two-fold or greater) and only a few (8 genes) were up-regulated (two-fold or greater). Interestingly, several down-regulated genes are also involved in other processes, such as inflammation and the immune response (TNF, TNFAIP2, TNFRSF1B, IL6R, CD27, LITAF, TNFRSF10C, and TNFSF8) or the response to stress (BCL6, NFKBIZ, and GADD45B). In contrast, in the latter group we found that 14 genes were differentially regulated (Table 3), and all of these genes were at least two-fold up-regulated. Numerous genes from this group are also involved in other processes, such as positive regulation of transcription and translation (NPM1, BCL3, and ATF6), positive regulation of I-kappaB kinase/NF-kappaB signaling (NPM1, BCL3, and CFLAR), signal transduction (NPM1, ATF6), positive regulation of protein kinase activity (JTB, PROK2), cellular homeostasis (MCL1), response to cytokine (MCL1), and angiogenesis (PROK2). Genes such as CDK6, CCND2, NPM1, TNFAIP3, JTB, PROK2, and CFLAR were also identified as having a role in positive regulation of the cell cycle, cell proliferation and cytoprotection. Next, we analyzed the expression of pro- or anti-apoptotic genes in GHD patients before GH-TS and compared them to controls, to check whether the lack of biological activity of endogenous GH may affect the expression of apoptosis-related genes. The global microarray analysis revealed that 20 pro- or anti-apoptotic genes were significantly altered in GH deficiency conditions (≥2.0-fold up- or down-regulation). Importantly, all genes of anti-apoptotic/pro-survival activity were down-regulated in GHD children before GH therapy (Table 4). Interestingly, the majority of the genes involved in the positive regulation of apoptosis were also down-regulated in GH deficiency state (Table 5). We found that some of these genes (CASP4, CASP8, and NFKBIZ) are also associated with the immune response. Moreover, several genes encoding the members of TNF and TNF receptor family (TNF, TNFRSF10C, TNFRSF1A, LITAF, and TNFAIP6) were also found to be down-regulated in GH deficiency. In contrast, one gene associated with execution phase of apoptosis, FNTA, was up-regulated in GH deficiency. Gene Ontology (GO) terms for biological processes associated with apoptosis and apoptosis-related mechanisms that are over-represented in the particular groups of significantly changed transcripts listed in the Table 2, Table 3, Table 4 and Table 5 are displayed in Supplementary Tables S1–S4, respectively. In summary, the increased expression of genes mostly associated with the negative regulation of apoptosis and pro-survival function was observed in CD34+ cells from GHD patients in course of GH-TS. Moreover, these genes were frequently down-regulated in GHD patients before GH therapy.

## 3. Discussion

In the last decade, GH therapy has yielded favorable results for childhood growth disorders and adult GHD; however, little is known about the particular functions in the hematopoietic system of GHD patients, including apoptosis, in course of GH therapy. GH initiates a cascade of biological effects by binding to the GH receptor (GHR), what induces direct phosphorylation of target signaling proteins ultimately leading to changes in gene expression [1]. Although a number of GH-regulated genes have been identified, genes relating GH to programmed cell death in hematopoietic stem and progenitor cells are not comprehensively known. Given that patients with GHD do not produce significant amounts of GH and GH therapy is the main source of hormone in their organism, we have chosen this disease as an in vivo model for identifying apoptosis-related genes that can be modulated in human HPCs by exogenous GH. Accordingly, we sought to investigate the effects of long-term GH treatment on apoptosis in hematopoietic system of children with severe GHD. The current study, to the best of our knowledge, is the first such study to evaluate the early and late apoptosis and its main control mechanisms in CD34+ cells collected from GHD children.

Apoptosis is a basic process, which contributes to the regulation of cell lifespan. In this notion, GH/GHR axis has been shown to be important regulator of apoptosis [19]. Apoptosis plays also a key role in the controlling of the hematopoietic cell homeostasis. Several hormones, such as prolactin or glucocorticoids have been shown to play a role in cell survival and apoptosis of the hematopoietic cells in vitro [20]. Moreover, we previously have found that thyroid hormones are able to affect apoptosis in normal human HPCs in vitro, and in patients suffering from hyperthyroidism [21,22]. There is a substantial empirical evidence to suggest that GH has also protective effect on human hematopoietic cells. This hypothesis is based on the observations of the GH-dependent increase in the number and production rate of different blood-borne cell populations in different cohorts of GHD patients treated with GH, including white or red blood cells (RBC). Christ et al. observed that a GH treatment over 3 months increased significantly the RBC counts in adult GHD patients [14]. Similarly, Bergamaschi et al. evaluated several GHD patients diagnosed with normochromic, normocytic anemia, and observed the increase in RBC counts, which restored their normal levels after 12 months of GH treatment [19]. The changes in RBC indices, hemoglobin concentration or hematopoietic precursor cell levels following GH therapy have also been observed in different groups of patients with and without GH deficiency [23,24,25]. Interestingly, when GH deficiency is associated with multiple pituitary hormone deficiencies, the pathological deficit in erythropoiesis was not corrected until GH treatment was started [15]. Likewise, the size of different leukocyte populations was significantly increased during GH administration, indicating an important role of GH in hematopoietic system functions [17,26,27]. We postulate here that the significantly decreased numbers of RBCs in GHD patients reported by other groups might be associated with the considerably higher percentage of apoptotic cells, and the increased BAX/BCL-2 mRNA and protein ratios, which were detected by us in CD34+ cells from untreated GHD patients compared to their controls. In contrast, diminished RBC deficiency in course of GH-TS may reflect either a stimulatory effect of GH on cell survival or/and inhibition of apoptosis mechanism. Therefore, it is crucial to analyze the involvement of GH in the regulation of apoptosis in CD34+ hematopoietic cells from GHD patients.

Several investigators reported that GHRs are expressed in diverse types of human mature hematopoietic cells, predominantly in monocytes/macrophages or B lymphocytes, but also in neutrophils, natural killer cells and T lymphocytes [28,29,30], suggesting a direct action of GH on these cells. Cool et al. also revealed that number of CD34+ cells in bone marrow of GHR deficient mice is markedly decreased relative to controls [31]. This may strongly indicate that GH has a potential influence on the development of hematopoietic progenitors in the bone marrow. However, there is lack of studies providing the evidence that active GHR is expressed in CD34+ cells circulating in GHD children. We demonstrated using IF technique that CD34+ cells from GHD patients express GHR protein on cell surface. Therefore, these cells are possible targets for GH-induced modulation of cellular functions. Similar results were reported by Gagnerault et al., who showed that all hematopoietic lineages of the murine bone marrow expressed GHR, however, at various levels [5]. We found that CD34+ cells from GHD children treated with GH for 3 months expressed significantly lower quantities of GHR protein compared to controls. This finding may be explained by the possibility that the produced GHR molecules were internalized into the cytoplasm in GH-activated cells as the form of the response to exogenously administered GH. In addition, the functional activity of GHR expressed in CD34+ cells was assessed. Our studies have shown that exogenous GH effectively activates GHR and the subsequent phosphorylation of STAT-5 protein. We also found that GHD state resulted in significant reduction of phosphorylated form of STAT-5 in CD34+ cells, which could be reversed by GH-TS. This result suggests that biological axis formed by exogenous GH and GHRs in CD34+ cells from GHD patients is fully functional and may induce the intracellular activation of GH/GHR-related signaling pathways during the long-term GH-TS, which therefore may contribute to the modulation of hematopoiesis in GHD patients.

In general, we found that CD34+ cells from GHD patients had different apoptosis rate compared to that of controls. Particularly, we demonstrated that these cells collected from GHD patients examined prior to the GH treatment exhibited considerably increased levels of apoptosis at both early and late phases compared to their controls. Similar results were reported by Kennedy et al., who showed an increased apoptosis rate in livers of Ames dwarf mice stably deficient in GH, suggesting that GH plays an important role in the cell survival [32]. Besides, Arnold and Weigent have shown that treatment of the lymphoma cells with antisense deoxyoligonucleotides to specifically block endogenous GH expression augmented cell apoptosis through the decreased expression of apoptosis inhibitors resulting in the DNA fragmentation [12]. In contrast, the treatment with GH resulted in a significant decrease of the percentage of apoptotic CD34+ cells from GHD patients in our study. Similar results were reported by Baixeras et al. [33] and by Haeffner et al. [8], who have confirmed that GH treatment can inhibit apoptosis in hematopoietic cells. GH has also been shown to be anti-apoptotic agent in other types of cells, including myoblasts, intestinal and ovarian granulosa cells [34,35,36]. The observed reduction of apoptosis due to the GH treatment may be explained by several mechanisms. The multiple actions of GH are initiated by binding to GHR. It activates the tyrosine kinase JAK-2, which is the major pathway whereby GH exerts its cellular effects [37]. The JAK-2-mediated signal transduction pathway has been shown to mediate induction of BCL-2 and NF-kB resulting in delay of hematopoietic cell death [6]. In addition to activating the RAS/MAPK pathway, GH can increase the homeo-box A1-dependent expression of BCL-2 as reported in human mammary carcinoma cells [38]. It is, therefore, conceivable that the up-regulation of the BCL-2 protein may be an essential mechanism by which GH directly exerts its protective effect on different cells. Likewise, we observed a significant increase in the Bcl-2 gene expression induced by GH therapy in cells of GHD patients, which was followed by clearly augmented production of Bcl-2 protein as detected by western blot analysis. In contrast, GH treatment did not affect the reported BCL-2 expression in blastocysts, but reduced the expression of the pro-apoptotic BAX protein [39]. Besides, GH treatment inhibited apoptosis in human leukemic cells and Chinese hamster ovary cells through stimulation of the anti-apoptotic serine kinase AKT, leaving unchanged the BCL-2 levels [40]. The GH-dependent activation of AKT has been shown to be dependent on the presence of the JAK-2 binding region in GHR molecule and was implicated in GH promotion of cell survival through inhibition of the pro-apoptotic caspase-3 [41]. In addition, GH increases the expression of STAT-5b in colonic epithelial cells, which might be involved in the down-regulation of pro-apoptotic peroxisome proliferator activated receptor-γ expression [42]. Besides, blocking endogenous GH action using a GHR antagonist has been associated with increased cell death [6]. Recently, Keane et al. proposed that observed GH induced anti-apoptotic effects may in part be mediated also through a pathway that alters the concentration of particular mitochondrially-associated miRNAs that can control the expression of apoptosis-related genes [43]. All the above mechanisms may simultaneously lead to observed GH-dependent anti-apoptotic effects in different types of cells, including CD34+ cells.

Apoptosis is regulated via the action of several oncogenes, and subsequently oncoproteins, which display inhibiting or promoting action. We demonstrated a strong relationship between the status of GH treatment and the expression of apoptosis-related genes such as anti-apoptotic BCL-2 and BCL-XL and the pro-apoptotic BAX gene. In particular, we investigated whether these genes with cell death/survival functions were differentially modulated by GH in hematopoietic CD34+ cells from GHD children. The BCL-2 gene encodes a protein that blocks programmed cell death without affecting cellular proliferation [44]. We found that CD34+ cells harvested from GH-treated patients exhibited significantly increased expression of both BCL-2 and BCL-XL genes at all examined time points of GH therapy. Our data are consistent with those obtained in previous studies and indicate that GH delivery to hematopoietic cells has a positive impact on the expression of selected anti-apoptotic genes in these cells [8,9,10]. The BAX protein is a member of the BCL-2 family that promotes apoptosis [45]. Here, the CD34+ cells from GHD patients examined prior to the GH treatment exhibited significantly increased expression of the BAX gene transcripts, and its corresponding BAX protein, compared to their controls. In our study, we surprisingly found that BAX gene was also significantly up-regulated in CD34+ cells from GHD patients treated with GH. This up-regulation might be a result of more rapid cellular turnover as well as an increased need for unnecessary cell elimination throughout hematopoietic lineage cell development, and both processes could be initially stimulated by exogenous GH. Additionally, this result may suggest that GH replacement during the first 6 months might be insufficient to block the pro-apoptotic mechanisms induced in GHD patients presenting the chronic endogenous GH deficiency conditions. On the other hand, the opposite results were reported by a number of authors, who demonstrated that expression of BAX gene together with other pro-apoptotic factors, such as BAD or caspases, were down-regulated in an in vitro model of T cell lymphoma over-expressing GH [12] and in cardiomyocytes [46] or colonocytes [47] of transgenic mice overexpressing GH gene as well as in myocytes [48] and neurons [49] of GH-treated animals. Unfortunately, at this stage of our research, it is impossible to define the exact mechanism of the observed BAX gene up-regulation during GH treatment. However, to evaluate whether GH treatment could potentially induce apoptosis via imbalance of BCL-2 and BAX expression in these cells, we performed quantitative analysis of BAX to BCL-2 ratio following GH treatment at mRNA and protein level. This ratio serves as a rheostat to determine the cell susceptibility to apoptosis, as increased BAX/BCL-2 ratio would favor cytochrome c release and activation of the mitochondrion-mediated signaling of apoptotic pathway with a final up-regulation of pro-apoptotic caspase-3 [45]. Therefore, we analyzed the BAX/BCL-2 mRNA and BAX/BCL-2 protein ratio in CD34+ cells from all the groups, and we found that GH therapy is associated with the pronounced reduction in the BAX/BCL-2 ratio at mRNA and protein level in 3rd and 6th month of GH-TS. This observation is in agreement with the earlier report [48] and with another finding of this study that GH therapy could significantly decrease the caspase-3/procaspase-3 ratio in cells from the GHD patients, as observed in both the 3rd and 6th month of GH treatment. Because caspase-3 exists as zymogen that must first be proteolytically cleaved to become activated protease, the caspase-3/procaspase-3 ratio has been used here as an index of caspase-3 activation in examined cells. In contrast, GHD patients examined prior to GH therapy demonstrated the increased BAX to BCL-2 ratio at both RNA and protein level compared to controls. Consequently, in untreated GHD patients, the apoptosis-associated caspase-3 activation was also significantly up-regulated.

Therefore, we decided to verify potential associations between systemic levels of GH and the expression of apoptosis-related genes in CD34+ cells from GHD patients comparing the genome-wide gene expression profiles of these cells collected from all the groups. We were interested in genes involved in such biological processes concerning induction and regulation of programmed cell death, extrinsic/intrinsic apoptotic signaling pathways, regulation of apoptotic signaling pathways, apoptosis-related nuclear and mitochondrial changes, cell-type specific apoptotic processes, execution phase of apoptosis and its regulation, regulation of enzyme activity involved in apoptotic process, regulation of hematopoietic cell apoptosis, and apoptotic signaling pathway in response to DNA damage. Comparison of the bioinformatics analyses of the complex gene datasets of examined cells identified that 6-month-GH treatment repressed significantly 25 pro-apoptotic genes and TNF was decreased the most. In parallel, the chronic GH administration significantly up-regulated only 8 pro-apoptotic genes. Since these genes are related not only to apoptosis induction, but also to pathways related to regulation of immune system processes and production of cytokines involved in the immune/stress responses of hematopoietic cells, the present study suggests that GH may play an inhibitory role on TNF-alpha production in human hematopoietic cells and therefore GH may also indirectly inhibit apoptosis by down-regulation of genes related to TNF-response signaling pathways. Our results seem to be in concert with the report of Andiran et al. [50], who found that in children with GHD, the TNF-alpha levels in PB decreased significantly during GH treatment, demonstrating a reverse correlation between TNF-alpha and GH concentrations in PB. Likewise, Serri et al. [51] examined the effect of GH deficiency and subsequent GH replacement on monocyte cytokine production and found that GH treatment of GHD patients led to a reduction in plasma TNF-alpha and IL-6 levels, and the decreased production of both cytokines from monocytes in vitro. Other studies showed that GH administration reduces circulating soluble apoptosis mediators, including TNF system components [52,53]. Altogether, our data suggest that GH may play a role in modulating the TNF-alpha expression in CD34+ cells. In contrast, among the anti-apoptotic/pro-survival genes with the largest expression change during GH therapy, all detected genes were up-regulated (14 genes), and our data suggest that GH is capable of effectively stimulate the anti-apoptotic signaling pathways. Interestingly, the most up-regulated gene in this group (almost 6 folds) was gene of cyclin-dependent kinase-6 (CDK6), which encodes serine/threonine-protein kinase involved in the control of the entrance into the cell cycle via interacting with D-type cyclins during G1 interphase and promotes G1/S transition in the cell cycle [54]. Concurrently, we found that CDK6-related cyclin D2 (CCND2) was also significantly up-regulated in CD34+ cells by GH therapy. Of note, CDK6 kinase is also implicated in initiation and maintenance of cell cycle exit during cell differentiation and is required for the proliferation of specific hematopoietic cell types (e.g., erythroid cells) [55], and it was reported that the TNF-alpha down-regulates CDK6 expression and induces apoptosis in human immature erythroid BFU-E cells [56]. In this notion, as GH administration significantly decreases TNF-alpha production, it would derepress CDK6 signaling pathway and this might be possible explanation how GH induces cell cycle and reversely inhibits apoptosis in hematopoietic cells. In addition, we found using microarray method the increased expression of several members of the BCL-2 gene family, such as BCL2A1 and BCL3, and these results corroborated our quantitative PCR data. This finding is also consistent with previous results obtained using human monocytic cells, promyelocytic leukemia U937 cell line or other immune-related cells [8,57]. Altogether, the increased gene expression of anti-apoptotic molecules in course of recombinant human GH therapy could be attributed to the activation of the body’s defense mechanisms to fight against the increased apoptosis rate, while insufficient amount of endogenous GH is present in GHD children prior to GH therapy. Especially, these data provide evidence that GH mediates its protective effect through enhancing the expression of the anti-apoptotic oncoprotein BCL-2 and other members of BCL-2-dependent pathways.

## 4. Materials and Methods

### 4.1. Subjects

We enrolled 40 children with height lower than the 3rd percentile on a growth chart, who were diagnosed with severe isolated GHD according to the established clinical criteria [58]. The GH-TS was performed by daily subcutaneous GH injections at 0.031 mg/kg/d. None of the patients suffered from diabetes insipidus, chromosomal abnormalities, dysmorphic syndromes, intestinal malabsorption, other chronic diseases or acquired GHD, as confirmed by a full clinical and laboratory evaluation. All subjects exhibited normal thyroid and adrenal function. Magnetic resonance imaging of hypothalamus and pituitary region was normal in all patients. 60 healthy children of similar ages, who did not differ significantly from GHD patients in terms of puberty and bone age, constituted the control group. The children’s pubertal stages were rated according to the Tanner method and are summarized in Table 1. All procedures were approved by the Local Ethics Committee of the Pomeranian Medical University and informed consent was provided for each patient.

### 4.2. Laboratory Measurements and Cell Isolation

PB samples were collected at the moment of GHD diagnosis and after 3 and 6 months of GH-TS and we determined hematological and hormonal parameters and isolated CD34+ cells. The selected hematological parameters were evaluated using cell analyzer (Cell Dyn 3000, Abbott Diagnostics, Mountain View, CA, USA). The mononuclear cell fraction was isolated by density gradient centrifugation and depleted of adherent and T cells. This fraction was next enriched for CD34+ cells using the CD34 MicroBead Kit (Miltenyi Biotech Inc., Auburn, CA, USA) according to the manufacturer’s protocol. Isolated CD34+ cells were next analyzed by flow cytometry to evaluate the efficiency of the CD34-positive sorting. CD34 antigen was expressed in 91.2% ± 4.8% of immunomagnetically isolated cells. The mean number of CD34+ cells collected from one subject during isolation procedure was 150,000 ± 105,000 cells in average.

### 4.3. Immunofluorescence Staining of PB-Derived CD34+ Cells

The CD34+ cells population was subjected to immunofluorescence staining for GHR. Briefly, isolated cells were fixed in 4% paraformaldehyde for 20 min and washed in PBS. GHR protein was detected using monoclonal PE-conjugated mouse anti-human GHR antibody (clone: MAB-1, Santa Cruz Biotechnology, Santa Cruz, CA, USA). CD45 protein was detected using FITC-conjugated anti-human monoclonal IgG (BD Biosciences, San Jose, CA, USA). Cells were subsequently labeled with DAPI (BD Biosciences) for nuclear staining. Fluorescent images were captured using Pathway Bioimager System (BD Biosciences).

### 4.4. Flow Cytometry

The CD34+ cells were quantitatively analyzed for the expression of GHR using flow cytometry. In particular, the mean fluorescence intensity (MFI) of GHR staining was assessed. Briefly, CD34+ cells were incubated (30 min) with mouse anti-human fluorochrome-conjugated monoclonal antibodies against specific antigens, including GHR, CD34, and CD45 (all from BD Biosciences). The cells were washed twice in ice-cold PBS, resuspended in 1% paraformaldehyde and analyzed in flow cytometer (LSRII, BD Biosciences) using the BD FACSDiva software.

### 4.5. Apoptosis Detection

The level of spontaneous apoptosis in CD34+ cells circulating in PB of GHD patients and controls was measured using two different methods. The Annexin V-FITC Apoptosis Detection Kit II (BD Biosciences) was used for the detection of early stage of apoptosis. To detect the late stage of apoptosis, the collected CD34+ cells were analyzed with the terminal deoxynucleotidyl transferase dUTP nick end labeling (TUNEL) assay using the APO-Direct Kit (BD Biosciences). Both kits were used according to the manufacturer’s instructions.

### 4.6. RNA Isolation and Gene Expression Analysis

Total mRNA was isolated from CD34+ cells using the RNeasy Mini Kit (Qiagen, Valencia, CA, USA). Subsequently, mRNA was reverse transcribed using the First Strand cDNA Synthesis Kit (Fermentas International Inc., Burlington, ON, Canada). Quantitative assessment of BCL-2, BCL-XL, BAX mRNA levels was performed using real time QRT-PCR carried out on a Bio-Rad CFX96 Real-Time PCR Detection System (Bio-Rad Inc., Hercules, CA, USA). The 25-µL reaction mixture contained 12.5 µL of SYBR Green PCR Master Mix, 10 ng of cDNA template, and one pair of the primers listed in Table 6. The relative quantification value of the target gene was normalized to the endogenous control gene of beta-2 microglobulin (BMG) and expressed as 2Δ*C*_t_, where Δ*C*_t_ = [*C*_t_ of BMG] − [*C*_t_ of target gene].

### 4.7. RNA Microarray Data Analysis

Total RNA was isolated from CD34+ cells using RNeasy Mini Kit (Qiagen). RNA was isolated from CD34+ cells of five GH-treated patients at baseline, and in 3rd and 6th month of treatment, and of five control subjects, and next was pooled to generate the final RNA sample representing a particular group of patients. Sense-strand cDNA generated from total RNA using an Ambion WT Expression Kit (Life Technologies, Paisley, UK) was fragmented and labeled using the GeneChipH WT Terminal Labeling Kit (Affymetrix, Santa Clara, CA, USA) and hybridized onto an Affymetrix WT Array Strip. Hybridization as well as subsequent fluidics and scanning steps were performed using an Affymetrix GeneAtlasTM system (Affymetrix). Differences in the expression of the chosen genes and Gene Ontology (GO) terms were analyzed in the R programming environment using Bioconductor packages.

### 4.8. ELISA

The systemic levels of IGF-1 were measured using commercially available, high-sensitivity ELISA Quantikine human immunoassay kit (R&D Systems, Minneapolis, MN, USA) according to the manufacturer’s instructions.

### 4.9. Western Blot Analysis

Total protein was isolated from CD34+ cells using PARIS kit (Life Technologies, Paisley, UK). Protein was isolated from CD34+ cells of seven GH-treated patients at baseline, and in 3rd and 6th month of treatment, and of seven control subjects, and next was pooled to generate the final protein sample representing a particular group of patients. Briefly, cells were homogenized in the Cell Disruption Buffer containing protease and phosphatase inhibitors (10 µg/mL leupeptin, 10 µg/mL aprotinin, 1 µg/mL pepstatin A, 1 mM sodium fluoride, and 2 mM Na3VO4) (all from Sigma Aldrich, St. Louis, MO, USA). The mixture was centrifuged, supernatants were collected, and protein concentrations were determined using the Bradford protein assay (Sigma Aldrich). Equal amounts of protein (20 µg/well) were loaded and separated by 4%–20% sodium dodecyl sulfate polyacrylamide gel electrophoresis (SDS-PAGE, mini-PROTEAN II electrophoresis system, Bio-Rad, Hercules, CA, USA) and then transferred to a 0.2-µm polyvinylidene fluoride (PVDF) membrane (Bio-Rad, Hercules, CA, USA). The membrane was probed with selected primary monoclonal/polyclonal IgG antibodies directed against the amino acid sequences of BCL-2, BAX, cleaved caspase-3, procaspase-3 and phosphorylated STAT-5 (all from Cell Signaling Technology, Beverly, MA, USA). Immunoreactive bands were detected using horseradish peroxidase-conjugated goat anti-rabbit and donkey-anti-goat secondary antibodies (Santa Cruz Biotechnology, Santa Cruz, CA, USA). Chemiluminescence detection was performed using the ECL Select Detection Kit (GE Healthcare, formerly Amersham Life Sciences, Little Chalfont, UK), and bands were subsequently visualized using a UVP camera (Gel DOC-It Imaging system; Bio-Rad, Hercules, CA, USA). Loading in the lanes was evaluated by stripping the blots for 2 h at 37 °C and then overnight at room temperature (IgG Elution Buffer; Thermo Fisher Scientific, Waltham, MA, USA). Reprobing was then performed in an analogous manner with a human anti-BMG monoclonal antibody (Santa Cruz Biotechnology, Santa Cruz, CA, USA) at a 1:1000 dilution, followed by an HRP-conjugated secondary antibody as described above. Protein levels were analyzed densitometrically by ImageJ software. Re-probing blots with more than one antibody against phosphorylated and total protein was performed in the same PVDF membranes. Each group analyzed in WB was performed in triplicate of biological replication.

### 4.10. Statistical Methods

Differences in the values of the quantitative parameters were compared between groups by unpaired Student *t*-test with Welch’s correction; for non-parametric tests, values were compared using the Mann-Whitney test. A *p* value of <0.05 was considered statistically significant.

## 5. Conclusions

This study used large-scale gene profiling via human RNA microarrays and sophisticated methods of apoptosis detection at different stages to monitor the apoptotic activity of CD34+ cells of hematopoietic origin in course of GH therapy. Three major conclusions can be drawn from our data: (i) GH treatment significantly decreased apoptosis in CD34+ cells. The analysis of Annexin V-stained cells and level of DNA fragmentation clearly demonstrated that GH therapy over 3 and 6 months is able to diminish apoptosis levels in these cells at its early and late phase. We herein reported that GH-dependent anti-apoptotic effect in hematopoietic cells is mediated by a significant increase in anti-apoptotic BCL-2 and BCL-xL gene expression, without effect on pro-apoptotic BAX gene; thus, the anti-apoptotic effects of GH may derive from a change in the BAX to BCL-2 ratio in these cells; (ii) The very interesting aspect of this study was visualizing the GHR expression in CD34+ cells indicating GHR availability and corresponding potential functionality of GH in hematopoietic progenitor cells from GHD patients. Moreover, we observed a chronic down-regulation of GHR protein at surface of CD34+ cells from GHD patients regardless of the undertaken GH treatment; (iii) Microarray data revealed that GH may have prevented apoptosis by altering stress-related TNF-alpha signaling since the expression of TNF-alpha and several members of TNF family was significantly decreased during GH administration.

## Figures and Tables

**Table 1:** Clinical characteristics of the study population.

Epidemiological and Clinical Characteristics	GHD Patients	Controls
Number of subjects/group (*n*)	40	60
Gender (male/female) (*n*)	26/12	43/17
	mean ± SD	mean ± SD
Mean age (years)	11 ± 4.2	13 ± 3.3
Mean BMI (units)	18.65 ± 3.5	19.17 ± 3.1
Plasma GH concentration (ng/mL) in different conditions:		
1. Mean nocturnal GH release	5.6 ± 2.7	NA
2. The oral clonidine provocative test	5.7 ± 2.4	NA
3. The L-Dopa provocative test	4.5 ± 2.2	NA
Plasma IGF-1 concentration (ng/mL)	125 ± 49.6 ^#^	162.3 ± 58.6
Plasma IGF-BP-3 concentration (µg/mL)	4.0 ± 1.6	4.15 ± 0.86
Sexual Maturity Rating system according to Tanner’s scale		
1. Tanner’s stage 1 (%)	60	60
2. Tanner’s stage 2 (%)	25	30
3. Tanner’s stage 3 (%)	15	10

# *p* < 0.05 vs. control group.

**Table 2:** The list of pro-apoptotic genes with the largest change of expression (at least 2 folds) in CD34+ cells from GHD patients treated for 6 months with GH-TS compared to GHD subjects before GH therapy.

No.	Gene Symbol	Gene Description	Entrez GeneID	Fold Change
1	TNF	tumor necrosis factor	7124	−3.5 *
2	TNFAIP2	tumor necrosis factor, alpha-induced protein 2	7127	−3.0
3	TNFRSF1B	tumor necrosis factor receptor superfamily, member 1B	7133	−3.0
4	SMNDC1	survival motor neuron domain containing 1	10285	−2.8
5	CD27	CD27 molecule	939	−2.4
6	BCL6	B-cell CLL/lymphoma 6	604	−2.4
7	NFKBIZ	nuclear factor of kappa light polypeptide gene enhancer in B-cells inhibitor, zeta	64332	−2.4
8	IL6R	interleukin 6 receptor	3570	−2.3
9	LITAF	lipopolysaccharide-induced TNF factor	9516	−2.3
10	TNFRSF10C	tumor necrosis factor receptor superfamily, member 10c	8794	−2.2
11	TNFSF8	tumor necrosis factor superfamily, member 8	944	−2.1
12	FOSB	FBJ murine osteosarcoma viral oncogene homolog B	2354	−2.0
13	FNTA	farnesyltransferase, CAAX box, alpha	2339	−2.0
14	DAP	death-associated protein	1611	−2.0
15	GADD45B	growth arrest and DNA-damage-inducible, beta	4616	−2.0
16	TMBIM1	transmembrane BAX inhibitor motif containing 1	64114	−2.0
17	FOXO3	forkhead box O3	2309	−2.0
18	ATM	ataxia telangiectasia mutated	472	−2.0
19	BID	BH3 interacting domain death agonist	637	−2.0
20	DEDD2	death effector domain containing 2	162989	−2.0
21	TRADD	TNFRSF1A-associated via death domain	8717	−2.0
22	MYD88	myeloid differentiation primary response gene (88)	4615	−2.0
23	PYCARD	PYD and CARD domain containing	29108	−2.0
24	TNFRSF1A	tumor necrosis factor receptor superfamily, member 1A	7132	−2.0
25	TNFRSF14	tumor necrosis factor receptor superfamily, member 14	8764	−2.0
26	TNFSF10	tumor necrosis factor superfamily, member 10	8743	2.0 ^#^
27	TNFSF13B	tumor necrosis factor superfamily, member 13b	10673	2.0
28	CASP1	caspase 1	834	2.0
29	CASP2	caspase 2	835	2.0
30	CASP4	caspase 4	837	2.0
31	DAPK1	death-associated protein kinase 1	1612	2.0
32	XAF1	XIAP associated factor 1	54739	2.9
33	TNFAIP6	tumor necrosis factor, alpha-induced protein 6	7130	4.8

* The negative value of Fold change indicates down-regulation of particular gene; ^#^ The positive value of Fold change indicates up-regulation of particular gene.

**Table 3:** The list of anti-apoptotic/pro-survival genes with the largest change of expression (at least 2 folds) in CD34+ cells from GHD patients treated for 6 months with GH-TS compared to GHD subjects before GH therapy.

No.	Gene Symbol	Gene Description	Entrez GeneID	Fold Change
1	CDK6	cyclin-dependent kinase 6	1021	5.7 ^#^
2	NPM1	nucleophosmin	4869	4.0
3	TNFAIP3	tumor necrosis factor, alpha-induced protein 3	7128	2.5
4	BCL2A1	BCL2-related protein A1	597	2.1
5	CCND2	cyclin D2	894	2.0
6	BCL3	B-cell CLL/lymphoma 3	602	2.0
7	JTB	jumping translocation breakpoint	10899	2.0
8	MCL1	myeloid cell leukemia sequence 1 (BCL2-related)	4170	2.0
9	PROK2	prokineticin 2	60675	2.0
10	PRDX5	peroxiredoxin 5	25824	2.0
11	DAD1	defender against cell death 1	1603	2.0
12	CFLAR	CASP8 and FADD-like apoptosis regulator	8837	2.0
13	ATF6	activating transcription factor 6	22926	2.0
14	CASP2	caspase 2	835	2.0

^#^ The positive value of Fold change indicates up-regulation of particular gene.

**Table 4:** The list of anti-apoptotic/pro-survival genes with the largest change of expression (at least 2 folds) in CD34+ cells from GHD patients prior to GH-TS compared to their controls.

No.	Gene Symbol	Gene Description	Entrez GeneID	Fold Change
1	BCL2A1	BCL2-related protein A1	597	−2.4 *
2	TNFAIP3	tumor necrosis factor, alpha-induced protein 3	7128	−2.4
3	BCL3	B-cell CLL/lymphoma 3	602	−2.1
4	ATF6	activating transcription factor 6	22926	−2.1
5	CFLAR	CASP8 and FADD-like apoptosis regulator	8837	−2.1
6	MCL1	myeloid cell leukemia sequence 1 (BCL2-related)	4170	−2.0
7	PROK2	prokineticin 2	60675	−2.0
8	PRDX5	peroxiredoxin 5	25824	−2.0
9	JTB	jumping translocation breakpoint	10899	−2.0

* The negative value of Fold change indicates down-regulation of particular gene.

**Table 5:** The list of pro-apoptotic genes with the largest change of expression (at least 2 folds) in CD34+ cells from untreated GHD patients prior to GH-TS compared to their controls.

No.	Gene Symbol	Gene Description	Entrez GeneID	Fold Change
1	FNTA	farnesyltransferase, CAAX box, alpha	2339	2.0 ^#^
2	NFKBIZ	nuclear factor of kappa light polypeptide gene enhancer in B-cells inhibitor, zeta	64332	−2.0 *
3	GADD45B	growth arrest and DNA-damage-inducible, beta	4616	−2.0
4	TNF	tumor necrosis factor	7124	−2.0
5	TNFRSF10C	tumor necrosis factor receptor superfamily, member 10c	8794	−2.0
6	TNFRSF1A	tumor necrosis factor receptor superfamily, member 1A	7132	−2.0
7	LITAF	lipopolysaccharide-induced TNF factor	9516	−2.0
8	CASP4	caspase 4	837	−2.0
9	CASP8	caspase 8	841	−2.0
10	TNFAIP6	tumor necrosis factor, alpha-induced protein 6	7130	−2.3
11	SMNDC1	survival motor neuron domain containing 1	10285	−4.1

* The negative value of Fold change indicates down-regulation of particular gene; ^#^ The positive value of Fold change indicates up-regulation of particular gene.

**Table 6:** List of primers used in this study.

No.	Gene Name	Gene Symbol	Primer Direction	Primer Sequence
1	B-cell CLL/lymphoma 2	***BCL-2***	Sense	GCC GGT TCA GGT ACT CAG TCA T
			Antisense	CAT GTG TGT GGA GAG CGT CAA
2	B-cell lymphoma-extra large	***BCL-XL***	Sense	CTC AGC GCT TGC TTT AC
			Antisense	CGC ACA GCA GCA GTT TGG
3	BCL2-associated X protein	***BAX***	Sense	GTT GCG GTC AGA AAA CAT GTC
			Antisense	GCC GCC GTG GAC ACA
4	Beta-2-microglobulin	***BMG***	Sense	AAT GCG GCA TCT TCA AAC CT
			Antisense	TGA CTT TGT CAC AGC CCA AGA TA

## Supplementary Materials

Supplementary materials can be found at www.mdpi.com/1422-0067/18/1/111/s1.

## References

[1] Brooks A.J., Waters M.J. (2010). The growth hormone receptor: Mechanism of activation and clinical implications. Nat. Rev. Endocrinol..

[2] Wang J., Zhou J., Cheng C.M., Kopchick J.J., Bondy C.A. (2004). Evidence supporting dual, IGF-I-independent and IGF-I-dependent, roles for GH in promoting longitudinal bone growth. J. Endocrinol..

[3] Sotiropoulos A., Ohanna M., Kedzia C., Menon R.K., Kopchick J.J., Kelly P.A., Pende M. (2006). Growth hormone promotes skeletal muscle cell fusion independent of insulin-like growth factor 1 up-regulation. Proc. Natl. Acad. Sci. USA.

[4] McLenachan S., Lum M.G., Waters M.J., Turnley A.M. (2009). Growth hormone promotes proliferation of adult neurosphere cultures. Growth Horm. IGF Res..

[5] Gagnerault M.C., Postel-Vinay M.C., Dardenne M. (1996). Expression of growth hormone receptors in murine lymphoid cells analyzed by flow cytometry. Endocrinology.

[6] Jeay S., Sonenshein G.E., Postel-Vinay M.C., Baixeras E. (2000). Growth hormone prevents apoptosis through activation of nuclear factor-kappaB in interleukin-3-dependent Ba/F3 cell line. Mol. Endocrinol..

[7] Matsuda T., Saito H., Inoue T., Fukatsu K., Han I., Furukawa S., Ikeda S., Muto T. (1998). Growth hormone inhibits apoptosis and up-regulates reactive oxygen intermediates production by human polymorphonuclear neutrophils. JPEN J. Parenter. Enter. Nutr..

[8] Haeffner A., Déas O., Mollereau B., Estaquier J., Mignon A., Haeffner-Cavaillon N., Harpentier B., Senik A., Hirsch F. (1999). Growth hormone prevents human monocytic cells from Fas-mediated apoptosis by up-regulating Bcl-2 expression. Eur. J. Immunol..

[9] Mitsunaka H., Dobashi H., Sato M., Tanaka T., Kitanaka A., Yamaoka G., Tokuda M., Matoba K., Hiraishi T., Ishida T. (2001). Growth hormone prevents Fas-induced apoptosis in lymphocytes through modulation of Bcl-2 and caspase-3. Neuroimmunomodulation.

[10] Lempereur L., Brambilla D., Scoto G.M., D’Alcamo M., Goffin V., Crosta L., Palmucci T., Rampello L., Bernardini R., Cantarella G. (2003). Growth hormone protects human lymphocytes from irradiation-induced cell death. Br. J. Pharmacol..

[11] Pulkki K.J. (1997). Cytokines and cardiomyocyte death. Ann. Med..

[12] Arnold R.E., Weigent D.A. (2004). The inhibition of apoptosis in EL4 lymphoma cells overexpressing growth hormone. Neuroimmunomodulation.

[13] Decker D., Springer W., Tolba R., Lauschke H., Hirner A., von Ruecker A. (2005). Perioperative treatment with human growth hormone down-regulates apoptosis and increases superoxide production in PMN from patients undergoing infrarenal abdominal aortic aneurysm repair. Growth Horm. IGF Res..

[14] Christ E.R., Cummings M.H., Westwood N.B., Sawyer B.M., Pearson T.C., Sönksen P.H., Russell-Jones D.L. (1997). The importance of growth hormone in the regulation of erythropoiesis, red cell mass, and plasma volume in adults with growth hormone deficiency. J. Clin. Endocrinol. Metab..

[15] Valerio G., di Maio S., Salerno M., Argenziano A., Badolato R., Tenore A. (1997). Assessment of red blood cell indices in growth-hormone-treated children. Horm. Res. Paediatr..

[16] Miniero R., Altomare F., Rubino M., Matarazzo P., Montanari C., Petri A., Raiola G., Bona G. (2012). Effect of recombinant human growth hormone (rhGH) on hemoglobin concentration in children with idiopathic growth hormone deficiency-related anemia. J. Pediatr. Hematol. Oncol..

[17] Sohmiya M., Kanazawa I., Kato Y. (2005). Effect of recombinant human GH on circulating granulocyte colony-stimulating factor and neutrophils in patients with adult GH deficiency. Eur. J. Endocrinol..

[18] Monson J.P. (2003). Long-term experience with GH replacement therapy: Efficacy and safety. Eur. J. Endocrinol..

[19] Bartke A., Sun L.Y., Longo V. (2013). Somatotropic signaling: Trade-offs between growth, reproductive development, and longevity. Physiol. Rev..

[20] Kiess W., Gallaher B. (1998). Hormonal control of programmed cell death/apoptosis. Eur. J. Endocrinol..

[21] Grymuła K., Paczkowska E., Dziedziejko V., Baśkiewicz-Masiuk M., Kawa M., Baumert B., Celewicz Z., Gawrych E., Machaliński B. (2007). The influence of 3,3′,5-triiodo-l-thyronine on human haematopoiesis. Cell Prolif..

[22] Kawa M.P., Grymula K., Paczkowska E., Baskiewicz-Masiuk M., Dabkowska E., Koziolek M., Tarnowski M., Kłos P., Dziedziejko V., Kucia M. (2010). Clinical relevance of thyroid dysfunction in human haematopoiesis: Biochemical and molecular studies. Eur. J. Endocrinol..

[23] Kotzmann H., Riedl M., Clodi M., Barnas U., Kaider A., Luger A. (1996). The influence of growth hormone substitution therapy on erythroid and myeloid progenitor cells and on peripheral blood cells in adult patients with growth hormone deficiency. Eur. J. Clin. Investig..

[24] Vihervuori E., Sipilä I., Siimes M.A. (1994). Increases in hemoglobin concentration and iron needs in response to growth hormone treatment. J. Pediatr..

[25] Meazza C., Bonomelli I., Pagani S., Travaglino P., Laarej K., Cantoni F., Bozzola M. (2009). Effect of human recombinant growth hormone therapy on circulating levels of EPO and G-CSF in short children. J. Pediatr. Endocrinol. Metab..

[26] Clark R. (1997). The somatogenic hormones and insulin-like growth factor-1: Stimulators of lymphopoiesis and immune function. Endocr. Rev..

[27] Goodier M.R., Imami N., Moyle G., Gazzard B., Gotch F. (2003). Loss of the CD56^hi^CD16^−^ NK cell subset and NK cell interferon-gamma production during antiretroviral therapy for HIV-1: Partial recovery by human growth hormone. Clin. Exp. Immunol..

[28] Rapaport R., Sills I.N., Green L., Barrett P., Labus J., Skuza K.A., Chartoff A., Goode L.A.U.R.A., Stene M.A.R.K., Petersen B.H. (1995). Detection of human growth hormone receptors on IM-9 cells and peripheral blood mononuclear cell subsets by flow cytometry: Correlation with growth hormone-binding protein levels. J. Clin. Endocrinol. Metab..

[29] Bresson J.L., Jeay S., Gagnerault M.C., Kayser C., Beressi N., Wu Z., Kinet S., Dardenne M., Postel-Vinay M.C. (1999). Growth hormone (GH) and prolactin receptors in human peripheral blood mononuclear cells: Relation with age and GH-binding protein. Endocrinology.

[30] Hattori N., Saito T., Yagyu T., Jiang B.H., Kitagawa K., Inagaki C. (2001). GH, GH receptor, GH secretagogue receptor, and ghrelin expression in human T cells, B cells, and neutrophils. J. Clin. Endocrinol. Metab..

[31] Cool S.M., Grünert M., Jackson R., Li H., Nurcombe V., Waters M.J. (2005). Role of growth hormone receptor signaling in osteogenesis from murine bone marrow progenitor cells. Biochem. Biophys. Res. Commun..

[32] Kennedy M.A., Rakoczy S.G., Brown-Borg H.M. (2003). Long-living Ames dwarf mouse hepatocytes readily undergo apoptosis. Exp. Gerontol..

[33] Baixeras E., Jeay S., Kelly P.A., Postel-Vinay M.C. (2001). The proliferative and antiapoptotic actions of growth hormone and insulin-like growth factor-1 are mediated through distinct signaling pathways in the Pro-B Ba/F3 cell line. Endocrinology.

[34] Segard H.B., Moulin S., Boumard S., Augier de Crémiers C., Kelly P.A., Finidori J. (2003). Autocrine growth hormone production prevents apoptosis and inhibits differentiation in C2C12 myoblasts. Cell Signal..

[35] Mylonas P.G., Matsouka P.T., Papandoniou E.V., Vagianos C., Kalfarentzos F., Alexandrides T.K. (2000). Growth hormone and insulin-like growth factor I protect intestinal cells from radiation induced apoptosis. Mol. Cell. Endocrinol..

[36] Sirotkin A.V., Makarevich A.V. (1999). GH regulates secretory activity and apoptosis in cultured bovine granulosa cells through the activation of the cAMP/protein kinase A system. J. Endocrinol..

[37] Argetsinger L.S., Campbell G.S., Yang X., Witthuhn B.A., Silvennoinen O., Ihle J.N., Carter-Su C. (1993). Identification of JAK2 as a growth hormone receptor-associated tyrosine kinase. Cell.

[38] Zhang X., Zhu T., Chen Y., Mertani H.C., Lee K.O., Lobie P.E. (2003). Human growth hormone-regulated HOXA1 is a human mammary epithelial oncogene. J. Biol. Chem..

[39] Kölle S., Stojkovic M., Boie G., Wolf E., Sinowatz F. (2002). Growth hormone inhibits apoptosis in in vitro produced bovine embryos. Mol. Reprod. Dev..

[40] Costoya J.A., Finidori J., Moutoussamy S., Seãris R., Devesa J., Arce V.M. (1999). Activation of growth hormone receptor delivers an antiapoptotic signal: Evidence for a role of Akt in this pathway. Endocrinology.

[41] Sanders E.J., Parker E., Harvey S. (2006). Retinal ganglion cell survival in development: Mechanisms of retinal growth hormone action. Exp. Eye Res..

[42] Bogazzi F., Ultimieri F., Raggi F., Russo D., Vanacore R., Guida C., Brogioni S., Cosci C., Gasperi M., Bartalena L. (2004). Growth hormone inhibits apoptosis in human colonic cancer cell lines: Antagonistic effects of peroxisome proliferator activated receptor-gamma ligands. Endocrinology.

[43] Keane J., Tajouri L., Gray B. (2015). The Effect of Growth Hormone Administration on the Regulation of Mitochondrial Apoptosis in-Vivo. Int. J. Mol. Sci..

[44] Hockenbery D., Nuñez G., Milliman C., Schreiber R.D., Korsmeyer S.J. (1990). Bcl-2 is an inner mitochondrial membrane protein that blocks programmed cell death. Nature.

[45] Oltvai Z.N., Milliman C.L., Korsmeyer S.J. (1993). Bcl-2 heterodimerizes in vivo with a conserved homolog, Bax, that accelerates programmed cell death. Cell.

[46] Bogazzi F., Russo D., Raggi F., Ultimieri F., Urbani C., Gasperi M., Bartalena L., Martino E. (2008). Transgenic mice overexpressing growth hormone (GH) have reduced or increased cardiac apoptosis through activation of multiple GH-dependent or -independent cell death pathways. Endocrinology.

[47] Bogazzi F., Ultimieri F., Raggi F., Russo D., Lombardi M., Cosci C., Brogioni S., Gasperi M., Bartalena L., Martino E. (2009). Reduced colonic apoptosis in mice overexpressing bovine growth hormone occurs through changes in several kinase pathways. Growth Horm. IGF Res..

[48] Dalla Libera L., Ravara B., Volterrani M., Gobbo V., Della Barbera M., Angelini A., Betto D.D., Germinario E., Vescovo G. (2004). Beneficial effects of GH/IGF-1 on skeletal muscle atrophy and function in experimental heart failure. Am. J. Physiol. Cell Physiol..

[49] Shin D.H., Lee E., Kim J.W., Kwon B.S., Jung M.K., Jee Y.H., Kim J., Bae S.R., Chang Y.P. (2004). Protective effect of growth hormone on neuronal apoptosis after hypoxia-ischemia in the neonatal rat brain. Neurosci. Lett..

[50] Andiran N., Yordam N. (2007). TNF-alpha levels in children with growth hormone deficiency and the effect of long-term growth hormone replacement therapy. Growth Horm. IGF Res..

[51] Serri O., St-Jacques P., Sartippour M., Renier G. (1999). Alterations of monocyte function in patients with growth hormone (GH) deficiency: Effect of substitutive GH therapy. J. Clin. Endocrinol. Metab..

[52] Parissis J.T., Adamopoulos S., Karatzas D., Paraskevaidis J., Livanis E., Kremastinos D. (2005). Growth hormone-induced reduction of soluble apoptosis mediators is associated with reverse cardiac remodelling and improvement of exercise capacity in patients with idiopathic dilated cardiomyopathy. Eur. J. Cardiovasc. Prev. Rehabil..

[53] Feldt-Rasmussen B., Lange M., Sulowicz W., Gafter U., Lai K.N., Wiedemann J., El Nahas M. (2007). Growth hormone treatment during hemodialysis in a randomized trial improves nutrition, quality of life, and cardiovascular risk. J. Am. Soc. Nephrol..

[54] Palmero I., Holder A., Sinclair A.J., Dickson C., Peters G. (1993). Cyclins D1 and D2 are differentially expressed in human B-lymphoid cell lines. Oncogene.

[55] Siebert R., Willers C.P., Opalka B. (1996). Role of the cyclin-dependent kinase 4 and 6 inhibitor gene family p15, p16, p18 and p19 in leukemia and lymphoma. Leuk. Lymphoma.

[56] Dai C., Chung I.J., Krantz S.B. (2004). In human immature BFU-E tumor necrosis factor-alpha not only downregulates CDK6 but also directly produces apoptosis which is prevented by stem cell factor. Exp. Hematol..

[57] Jeay S., Sonenshein G.E., Postel-Vinay M.C., Kelly P.A., Baixeras E. (2002). Growth hormone can act as a cytokine controlling survival and proliferation of immune cells: New insights into signaling pathways. Mol. Cell. Endocrinol..

[58] Growth Hormone Research Society (2000). Consensus guidelines for the diagnosis and treatment of growth hormone (GH) deficiency in childhood and adolescence: Summary statement of the GH Research Society. J. Clin. Endocrinol. Metab..

