# Supplementary material for: The Impact of Growth Hormone Therapy on the Apoptosis Assessment in CD34+ Hematopoietic Cells from Children with Growth Hormone Deficiency
Source: Int J Mol Sci. 2017 Jan 7;18(1):111. doi: 10.3390/ijms18010111 (PMC5297745; doi:10.3390/ijms18010111)
Supplement: Supplementary file 1 [file ijms-18-00111-s001.pdf]

Supplementary Materials: The Impact of Growth Hormone Therapy on the Apoptosis Assessment in CD34+ Hematopoietic Cells from Children with Growth Hormone Deficiency

Miłosz Piotr Kawa, Iwona Stecewicz, Katarzyna Piecyk, Edyta Paczkowska, Dorota Rogińska, Anna Sobuś, Karolina Łuczkowska, Ewa Pius-Sadowska, Elżbieta Gawrych, Elżbieta Petriczko, Mieczysław Walczak and Bogusław Machaliński

Table S1. Gene Ontology (GO) terms for apoptosis-related biological processes that are over-represented in the list of significantly changed pro-apoptotic genes in the Table 2 in $CD34^{+}$ cells from GHD patients treated for 6 months with GH-TS compared to GHD subjects before GH therapy.

Human Gene	GO ID $	Qualified GO Term
TNF	GO:0043068	positive regulation of programmed cell death
	GO:0071550	death-inducing signaling complex assembly
	GO:0008625	extrinsic apoptotic signaling pathway via death domain receptors
	GO:0008630	intrinsic apoptotic signaling pathway in response to DNA damage
	GO:0006919	activation of cysteine-type endopeptidase activity involved in apoptotic process
	GO:0006927	transformed cell apoptotic process
TNFAIP2	GO:0001525	angiogenesis
TNFRSF1B	GO:0097190	apoptotic signaling pathway
	GO:0097191	extrinsic apoptotic signaling pathway
	GO:0007568	aging
	GO:0008630	intrinsic apoptotic signaling pathway in response to DNA damage
	GO:0032496	response to lipopolysaccharide
	GO:0006954	inflammatory response
	GO:0050779	RNA destabilization
	GO:0007275	multicellular organism development
	GO:0042127	regulation of cell proliferation
CD27	GO:0006954	inflammatory response
	GO:0097191	extrinsic apoptotic signaling pathway
	GO:0070233	negative regulation of T cell apoptotic process
	GO:0008588	release of cytoplasmic sequestered NF-kappaB
	GO:0008625	extrinsic apoptotic signaling pathway via death domain receptors
	GO:0043154	negative regulation of cysteine-type endopeptidase activity involved in apoptotic process
	GO:0032496	response to lipopolysaccharide
	GO:0006955	immune response
	GO:0007166	cell surface receptor signaling pathway
BCL6	GO:0042127	regulation of cell proliferation
	GO:0042981	regulation of apoptotic process
	GO:0006974	cellular response to DNA damage stimulus
	GO:0002903	negative regulation of B cell apoptotic process
	GO:0030308	negative regulation of cell growth
	GO:0043066	negative regulation of apoptotic process
	GO:0048821	erythrocyte development
	GO:0030183	B cell differentiation
	GO:0030099	myeloid cell differentiation
	GO:0006954	inflammatory response
	GO:0002829	negative regulation of type 2 immune response
	GO:0050776	regulation of immune response
	GO:2000773	negative regulation of cellular senescence
NFKBIZ	GO:0006351	transcription, DNA-templated

Table S1. *Cont.*

Human Gene	GO ID [$]	Qualified GO Term
IL6R	GO:0019221	cytokine-mediated signaling pathway
	O:0006953	acute-phase response
	GO:0010536	positive regulation of activation of Janus kinase activity
	GO:0042517	positive regulation of tyrosine phosphorylation of Stat3 protein
	GO:0002548	monocyte chemotaxis
	GO:0002690	positive regulation of leukocyte chemotaxis
	GO:0019221	cytokine-mediated signaling pathway
	GO:0048661	positive regulation of smooth muscle cell proliferation
	GO:0051092	negative regulation of NF-kappaB transcription factor activity
LITAF	GO:0006351	transcription, DNA-templated
	GO:0007165	signal transduction
	GO:0007568	cell aging
	GO:0042347	negative regulation of NF-kappaB import into nucleus
	GO:0043123	positive regulation of I-kappaB kinase/NF-kappaB signaling
TNFRSF10C	GO:0008625	extrinsic apoptotic signaling pathway via death domain receptors
	GO:0033209	tumor necrosis factor-mediated signaling pathway
	GO:0007275	multicellular organism development
	GO:0032496	response to lipopolysaccharide
	GO:0007165	signal transduction
TNFSF8	GO:0006915	apoptotic process
	GO:0007165	signal transduction
FOSB	GO:0000122	negative regulation of transcription from RNA polymerase II promoter
	GO:0006355	regulation of transcription, DNA-templated
	GO:0042493	response to drug
	GO:0045944	positive regulation of transcription from RNA polymerase II promoter
	GO:0071277	cellular response to calcium ion
FNTA	GO:0012501	programmed cell death
	GO:0006921	cellular component disassembly involved in execution phase of apoptosis
	GO:0007528	neuromuscular junction development
DAP	GO:0006915	apoptotic process
	GO:0097190	apoptotic signaling pathway
	GO:0006914	autophagy
	GO:0010507	negative regulation of autophagy
	GO:0006919	activation of cysteine-type endopeptidase activity involved in apoptotic process
	GO:0045892	negative regulation of transcription, DNA-templated
	GO:0032088	negative regulation of NF-kappaB transcription factor activity
GADD45B	GO:0006915	apoptotic process
	GO:0006950	response to stress
	GO:0006469	negative regulation of protein kinase activity
	GO:0000186	activation of MAPKK activity
	GO:0000185	activation of MAPKKK activity
	GO:1900745	positive regulation of p38MAPK cascade
	GO:0046330	positive regulation of JNK cascade
	GO:0051726	regulation of cell cycle
	GO:0007275	multicellular organism development
TMBIM1	GO:1902042	regulation of extrinsic apoptotic signaling pathway via DDR
	GO:1902045	regulation of Fas signaling pathway
FOXO3	GO:0033209	tumor necrosis factor-mediated signaling pathway
	GO:0034599	cellular response to oxidative stress
	GO:0030330	DNA damage response, signal transduction by p53 class mediator
	GO:0006355	regulation of transcription, DNA-templated
	GO:0006357	regulation of transcription from RNA polymerase II promoter
	GO:0006366	transcription from RNA polymerase II promoter
	GO:0090090	negative regulation of canonical Wnt signaling pathway
	GO:0006417	regulation of translation
	GO:0030154	cell differentiation
	GO:0045648	regulation of erythrocyte differentiation
	GO:0007173	epidermal growth factor receptor signaling pathway

Table S1. *Cont.*

Human Gene	GO ID $	Qualified GO Term
ATM	GO:0006915	apoptotic process
	GO:0051402	neuron apoptotic process
	GO:0008630	intrinsic apoptotic signaling pathway in response to DNA damage
	GO:0043525	positive regulation of neuron apoptotic process
	GO:1901216	positive regulation of neuron death
	GO:0090399	replicative senescence
	GO:0001666	response to hypoxia
	GO:0010506	regulation of autophagy
	GO:0034605	cellular response to heat
	GO:0006974	cellular response to DNA damage stimulus
	GO:0006281	DNA repair
	GO:0006975	DNA damage induced protein phosphorylation
	GO:0006977	DNA damage response, signal transduction by p53 resulting in cell cycle arrest
	GO:0006302	double-strand break repair
	GO:0045003	double-strand break repair via synthesis-dependent strand annealing
	GO:0043517	positive regulation of DNA damage response, signal transduction by p53
	GO:0051726	regulation of cell cycle
	GO:0007050	cell cycle arrest
	GO:0007165	signal transduction
	GO:1901796	regulation of signal transduction by p53 class mediator
	GO:0006468	protein phosphorylation
	GO:0046777	protein autophosphorylation
	GO:0010212	response to ionizing radiation
	GO:0071480	cellular response to gamma radiation
	GO:0071500	cellular response to nitrosative stress
BID	GO:0006915	apoptotic process
	GO:0006919	activation of cysteine-type endopeptidase activity involved in apoptotic process
	GO:0008625	extrinsic apoptotic signaling pathway via death domain receptors
	GO:0097193	intrinsic apoptotic signaling pathway
	GO:2001244	positive regulation of intrinsic apoptotic signaling pathway
	GO:0097191	extrinsic apoptotic signaling pathway
	GO:2001238	positive regulation of extrinsic apoptotic signaling pathway
	GO:0042770	signal transduction in response to DNA damage
	GO:0042127	regulation of cell proliferation
	GO:0051402	neuron apoptotic process
	GO:0034349	glial cell apoptotic process
	GO:0008637	apoptotic mitochondrial changes
	GO:0090200	positive regulation of release of cytochrome c from mitochondria
	GO:0097345	mitochondrial outer membrane permeabilization
	GO:1900740	regulation of protein insertion into mitochondrial membrane involved in apoptotic signaling pathway
	GO:1901030	regulation of mitochondrial outer membrane permeabilization involved in apoptotic signaling pathway
DEDD2	GO:0008625	extrinsic apoptotic signaling pathway via death domain receptors
	GO:2001238	positive regulation of extrinsic apoptotic signaling pathway
	GO:0030262	apoptotic nuclear changes
	GO:0035556	intracellular signal transduction
	GO:0006351	transcription, DNA-templated
TRADD	GO:0006915	apoptotic process
	GO:0012501	programmed cell death
	GO:0097191	extrinsic apoptotic signaling pathway
	GO:1902041	regulation of extrinsic apoptotic signaling pathway via DDR
	GO:0097296	activation of cysteine-type endopeptidase activity involved in apoptotic signaling pathway
	GO:0006919	activation of cysteine-type endopeptidase activity involved in apoptotic process
	GO:0043154	negative regulation of cysteine-type endopeptidase activity involved in apoptotic process
	GO:0010803	regulation of tumor necrosis factor-mediated signaling pathway
	GO:0007165	signal transduction
	GO:0007249	I-kappaB kinase/NF-kappaB signaling

Table S1. *Cont.*

Human Gene	GO ID $	Qualified GO Term
MYD88	GO:0043066	regulation of apoptotic process
	GO:0007165	signal transduction
	GO:0007254	JNK cascade
	GO:0019221	cytokine-mediated signaling pathway
	GO:0006954	inflammatory response
	GO:0050727	regulation of inflammatory response
	GO:2000338	regulation of chemokine (C-X-C motif) ligand 1 production
	GO:1902622	regulation of neutrophil migration
	GO:0051092	regulation of NF-kappaB transcription factor activity
	GO:0032496	response to lipopolysaccharide
	GO:0031663	lipopolysaccharide-mediated signaling pathway
	GO:0032680	regulation of tumor necrosis factor production
	GO:0032760	positive regulation of tumor necrosis factor production
	GO:0032755	positive regulation of interleukin-6 production
	GO:0032747	positive regulation of interleukin-23 production
	GO:0070555	response to interleukin-1
	GO:0042127	regulation of cell proliferation
	GO:0050671	regulation of lymphocyte proliferation
	GO:0048661	regulation of smooth muscle cell proliferation
	GO:0071260	cellular response to mechanical stimulus
PYCARD	GO:0042981	regulation of apoptotic process
	GO:0043065	positive regulation of apoptotic process
	GO:0042771	intrinsic apoptotic signaling pathway in response to DNA damage by p53
	GO:2001238	positive regulation of extrinsic apoptotic signaling pathway
	GO:0072332	intrinsic apoptotic signaling pathway by p53 class mediator
	GO:2001242	regulation of intrinsic apoptotic signaling pathway
	GO:0006954	inflammatory response
	GO:0071222	cellular response to lipopolysaccharide
	GO:0010803	regulation of tumor necrosis factor-mediated signaling pathway
	GO:0032760	positive regulation of tumor necrosis factor production
	GO:0033209	tumor necrosis factor-mediated signaling pathway
	GO:0071356	cellular response to tumor necrosis factor
	GO:0050718	positive regulation of interleukin-1 beta secretion
	GO:0032755	positive regulation of interleukin-6 production
	GO:2000778	positive regulation of interleukin-6 secretion
	GO:0032729	positive regulation of interferon-gamma production
	GO:0007165	signal transduction
	GO:0070374	regulation of ERK1 and ERK2 cascade
	GO:0071901	negative regulation of protein serine/threonine kinase activity
	GO:0051091	positive regulation of sequence-specific DNA binding transcription factor activity
	GO:0051092	positive regulation of NF-kappaB transcription factor activity
	GO:0043124	regulation of I-kappaB kinase/NF-kappaB signaling
TNFRSF1A	GO:0008625	extrinsic apoptotic signaling pathway via death domain receptors
	GO:0008630	intrinsic apoptotic signaling pathway in response to DNA damage
	GO:0071550	death-inducing signaling complex assembly
	GO:0006954	inflammatory response
	GO:0050728	regulation of inflammatory response
	GO:0007165	signal transduction
	GO:0007249	I-kappaB kinase/NF-kappaB signaling
	GO:0007166	cell surface receptor signaling pathway
	GO:0019221	cytokine-mediated signaling pathway
	GO:0042127	regulation of cell proliferation
	GO:0007275	multicellular organism development
TNFRSF14	GO:0097190	apoptotic signaling pathway
	GO:0007166	cell surface receptor signaling pathway
	GO:0042127	regulation of cell proliferation
	GO:0043410	regulation of MAPK cascade
	GO:0046642	regulation of T cell proliferation
	GO:0007275	multicellular organism development

Table S1. *Cont.*

Human Gene	GO ID $	Qualified GO Term
TNFSF10	GO:0012501	programmed cell death
	GO:2001238	regulation of extrinsic apoptotic signaling pathway
	GO:1902041	regulation of extrinsic apoptotic signaling pathway via death domain receptors
	GO:1902042	negative regulation of extrinsic apoptotic signaling pathway via DDR
	GO:0006919	activation of cysteine-type endopeptidase activity involved in apoptotic process
	GO:0097296	activation of cysteine-type endopeptidase activity involved in apoptotic signaling pathway
	GO:0043154	negative regulation of cysteine-type endopeptidase activity involved in apoptotic process
	GO:0070266	necroptotic process
	GO:0010939	regulation of necrotic cell death
	GO:0097300	programmed necrotic cell death
	GO:0007165	signal transduction
	GO:0007267	cell-cell signaling
TNFSF13B	GO:0006955	immune response
CASP1	GO:0006919	activation of cysteine-type endopeptidase activity involved in apoptotic process
	GO:0007165	signal transduction
	GO:0043123	positive regulation of I-kappaB kinase/NF-kappaB signaling
	GO:0050715	positive regulation of cytokine secretion
	GO:0032611	interleukin-1 beta production
	GO:0050717	positive regulation of interleukin-1 alpha secretion
	GO:0050718	positive regulation of interleukin-1 beta secretion
	GO:0032496	response to lipopolysaccharide
	GO:0001666	response to hypoxia
	GO:0016485	protein processing
CASP2	GO:0006915	apoptotic process
	GO:0097194	execution phase of apoptosis
	GO:2001235	regulation of apoptotic signaling pathway
	GO:0006919	activation of cysteine-type endopeptidase activity involved in apoptotic process
	GO:0043281	regulation of cysteine-type endopeptidase activity involved in apoptotic process
	GO:0035234	ectopic germ cell programmed cell death
	GO:0006977	DNA damage response, signal transduction by p53 resulting in cell cycle arrest
	GO:0016485	protein processing
	GO:0006508	proteolysis
	GO:0007568	cell aging
CASP4	GO:0097193	intrinsic apoptotic signaling pathway
	GO:0070059	intrinsic apoptotic signaling pathway in response to RE stress
	GO:0050727	regulation of inflammatory response
	GO:1904646	cellular response to beta-amyloid
	GO:0006508	proteolysis
DAPK1	GO:0006915	apoptotic process
	GO:004306	negative regulation of apoptotic process
	GO:009710	apoptotic signaling pathway
	GO:009791	extrinsic apoptotic signaling pathway
	GO:0008625	extrinsic apoptotic signaling pathway via death domain receptors
	GO:1902042	negative regulation of extrinsic apoptotic signaling pathway via DDR
	GO:0097192	extrinsic apoptotic signaling pathway in absence of ligand
	GO:0071346	cellular response to interferon-gamma
	GO:0007165	signal transduction
	GO:0035556	intracellular signal transduction
	GO:0006468	protein phosphorylation
	GO:0046777	protein autophosphorylation
XAF1	GO:0006915	apoptotic process
TNFAIP6	GO:0007165	signal transduction
	GO:0007267	cell-cell signaling

$ Data from the Gene Ontology (2009).

Table S2. Gene Ontology (GO) terms for cell survival-related biological processes that are over-represented in the list of significantly changed anti-apoptotic/pro-survival genes in the Table 3 in CD34+ cells from GHD patients treated for 6 months with GH-TS compared to GHD subjects before GH therapy.

Human Gene	GO ID $	Qualified GO Term
CDK6	GO:0060218	hematopoietic stem cell differentiation
	GO:0002244	hematopoietic progenitor cell differentiation
	GO:0045596	regulation of cell differentiation
	GO:0045646	regulation of erythrocyte differentiation
	GO:0033077	T cell differentiation in thymus
	GO:0045638	regulation of myeloid cell differentiation
	GO:0000082	G1/S transition of mitotic cell cycle
	GO:0051301	cell division
	GO:0007050	control of cell cycle arrest
	GO:0010468	regulation of gene expression
	GO:0010628	positive regulation of gene expression
	GO:0006468	protein phosphorylation
	GO:0007219	Notch signaling pathway
	GO:0048699	generation of neurons
	GO:0042063	gliogenesis
	GO:0050680	negative regulation of epithelial cell proliferation
	GO:2000773	negative regulation of cellular senescence
	GO:0001954	positive regulation of cell-matrix adhesion
NPM1	GO:0043066	negative regulation of apoptotic process
	GO:0044387	negative regulation of kinase activity by regulation of protein phosphorylation
	GO:1902751	regulation of cell cycle G2/M phase transition
	GO:0008104	protein localization
	GO:0051092	positive regulation of NF-kappaB transcription factor activity
	GO:0045727	positive regulation of translation
	GO:0060735	regulation of eIF2 alpha phosphorylation by dsRNA
	GO:0060699	regulation of endoribonuclease activity
	GO:0007569	cell aging
TNFAIP3	GO:0006915	apoptotic process
	GO:0060548	negative regulation of cell death
	GO:1902042	negative regulation of extrinsic apoptotic signaling pathway via DDR
	GO:2000352	negative regulation of endothelial cell apoptotic process
	GO:0033209	tumor necrosis factor-mediated signaling pathway
	GO:0010803	regulation of tumor necrosis factor-mediated signaling pathway
	GO:2000349	negative regulation of CD40 signaling pathway
	GO:0050727	regulation of inflammatory response
	GO:0050728	negative regulation of inflammatory response
	GO:0002677	negative regulation of chronic inflammatory response
	GO:0032691	negative regulation of interleukin-1 beta production
	GO:0032703	negative regulation of interleukin-2 production
	GO:0032715	negative regulation of interleukin-6 production
	GO:0045736	regulation of cyclin-dependent protein serine/threonine kinase activity
	GO:0032088	negative regulation of NF-kappaB transcription factor activity
	GO:0043124	negative regulation of I-kappaB kinase/NF-kappaB signaling
	GO:0006955	immune response
	GO:2000347	positive regulation of hepatocyte proliferation
	GO:0090291	regulation of osteoclast proliferation
	GO:0070301	cellular response to hydrogen peroxide
	GO:0051259	protein oligomerization
BCL2A1	GO:0043066	negative regulation of apoptotic process
	GO:0008630	intrinsic apoptotic signaling pathway in response to DNA damage
	GO:0001836	release of cytochrome c from mitochondria
CCND2	GO:0000079	regulation of cyclin-dependent protein serine/threonine kinase activity
	GO:0050679	positive regulation of epithelial cell proliferation
	GO:0045737	positive regulation of cyclin-dependent protein kinase activity
	GO:0008284	positive regulation of cell proliferation
	GO:0051726	regulation of cell cycle
	GO:0001934	positive regulation of protein phosphorylation
	GO:0000082	G1/S transition of mitotic cell cycle
	GO:0051301	regulation of cell division

Table S2. *Cont.*

Human Gene	GO ID $	Qualified GO Term
BCL3	GO:0006351	transcription, DNA-templated
	GO:0045893	positive regulation of transcription, DNA-templated
	GO:0045944	positive regulation of transcription from RNA polymerase II promoter
	GO:0045727	positive regulation of translation
	GO:0007249	I-kappaB kinase/NF-kappaB signaling
	GO:0006974	cellular response to DNA damage stimulus
	GO:0030330	DNA damage response, signal transduction by p53 class mediator
	GO:0045064	T-helper cell differentiation
	GO:0002315	marginal zone B cell differentiation
	GO:0045415	negative regulation of interleukin-8 biosynthetic process
	GO:0042536	negative regulation of tumor necrosis factor biosynthetic process
	GO:0032729	positive regulation of interferon-gamma production
	GO:0045082	positive regulation of interleukin-10 biosynthetic process
	GO:0051457	maintenance of protein location in nucleus
JTB	GO:0008637	apoptotic mitochondrial changes
MCL1	GO:1903378	positive regulation of oxidative stress-induced neuron intrinsic apoptosis
	GO:0097192	extrinsic apoptotic signaling pathway in absence of ligand
	GO:0034097	response to cytokine
	GO:0007275	multicellular organism development
	GO:0019725	cellular homeostasis
PROK2	GO:0043066	negative regulation of apoptotic process
	GO:0001525	angiogenesis
	GO:0045765	regulation of angiogenesis
	GO:0007186	G-protein coupled receptor signaling pathway
	GO:0007218	neuropeptide signaling pathway
	GO:0006954	inflammatory response
	GO:0006935	chemotaxis
PRDX5	GO:0006915	apoptotic process
	GO:0043066	negative regulation of apoptotic process
	GO:0034614	cellular response to reactive oxygen species
	GO:0051354	negative regulation of oxidoreductase activity
	GO:0070995	NADPH oxidation
DAD1	GO:0044267	cellular protein metabolic process
	GO:0043687	post-translational protein modification
	GO:0006486	protein glycosylation
CFLAR	GO:0006915	apoptotic process
	GO:0012501	programmed cell death
	GO:0043066	negative regulation of apoptotic process
	GO:0043154	negative regulation of cysteine-type endopeptidase activity involved in apoptosis
	GO:2001237	negative regulation of extrinsic apoptotic signaling pathway
	GO:1902041	regulation of extrinsic apoptotic signaling pathway via DDR
	GO:0097296	activation of cysteine-type endopeptidase activity involved in apoptosis
	GO:0097194	execution phase of apoptosis
	GO:0010939	regulation of necrotic cell death
	GO:0051092	positive regulation of NF-kappaB transcription factor activity
	GO:0007519	skeletal muscle tissue development
	GO:0014842	regulation of skeletal muscle satellite cell proliferation
	GO:0043403	skeletal muscle tissue regeneration
ATF6	GO:0007165	signal transduction
	GO:0006990	positive regulation of transcription from RNA polymerase II promoter
	GO:0044267	cellular protein metabolic process

Table S2. *Cont.*

Human Gene	GO ID $	Qualified GO Term
CASP2	GO:0006915	apoptotic process
	GO:0097194	execution phase of apoptosis
	GO:2001235	regulation of apoptotic signaling pathway
	GO:0006919	activation of cysteine-type endopeptidase activity involved in apoptotic process
	GO:0043281	regulation of cysteine-type endopeptidase activity involved in apoptotic process
	GO:0035234	ectopic germ cell programmed cell death
	GO:0006977	DNA damage response, signal transduction by p53 resulting in cell cycle arrest
	GO:0016485	protein processing
	GO:0006508	proteolysis
	GO:0007568	aging

$ Data from the Gene Ontology (2009).

Table S3. Gene Ontology (GO) terms for cell survival-related biological processes that are over-represented in the list of significantly changed anti-apoptotic/pro-survival genes in the Table 4 in $CD34^+$ cells from untreated GHD patients prior to GH-TS compared to their controls

Human Gene	GO ID $	Qualified GO Term
BCL2A1	GO:0043066	negative regulation of apoptotic process
	GO:0008630	intrinsic apoptotic signaling pathway in response to DNA damage
	GO:0001836	release of cytochrome c from mitochondria
TNFAIP3	GO:0006915	apoptotic process
	GO:0060548	negative regulation of cell death
	GO:1902042	negative regulation of extrinsic apoptotic pathway via DDR
	GO:2000352	negative regulation of endothelial cell apoptotic process
	GO:0033209	tumor necrosis factor-mediated signaling pathway
	GO:0010803	regulation of tumor necrosis factor-mediated signaling pathway
	GO:2000349	negative regulation of CD40 signaling pathway
	GO:0050727	regulation of inflammatory response
	GO:0050728	negative regulation of inflammatory response
	GO:0002677	negative regulation of chronic inflammatory response
	GO:0032691	negative regulation of interleukin-1 beta production
	GO:0032703	negative regulation of interleukin-2 production
	GO:0032715	negative regulation of interleukin-6 production
	GO:0045736	regulation of cyclin-dependent protein serine/threonine kinase activity
	GO:0032088	negative regulation of NF-kappaB transcription factor activity
	GO:0043124	negative regulation of I-kappaB kinase/NF-kappaB signaling
	GO:0006955	immune response
	GO:2000347	positive regulation of hepatocyte proliferation
	GO:0090291	regulation of osteoclast proliferation
	GO:0070301	cellular response to hydrogen peroxide
	GO:0051259	protein oligomerization
BCL3	GO:0006351	transcription, DNA-templated
	GO:0045893	positive regulation of transcription, DNA-templated
	GO:0045944	positive regulation of transcription from RNA polymerase II promoter
	GO:0045727	positive regulation of translation
	GO:0007249	I-kappaB kinase/NF-kappaB signaling
	GO:0006974	cellular response to DNA damage stimulus
	GO:0030330	DNA damage response, signal transduction by p53 class mediator
	GO:0045064	T-helper cell differentiation
	GO:0002315	marginal zone B cell differentiation
	GO:0045415	negative regulation of interleukin-8 biosynthetic process
	GO:0042536	negative regulation of tumor necrosis factor biosynthetic process
	GO:0032729	positive regulation of interferon-gamma production
	GO:0045082	positive regulation of interleukin-10 biosynthetic process
	GO:0051457	maintenance of protein location in nucleus
ATF6	GO:0007165	signal transduction
	GO:0006990	positive regulation of transcription from RNA polymerase II promoter
	GO:0044267	cellular protein metabolic process

Table S3. *Cont.*

Human Gene	GO ID $	Qualified GO Term
CFLAR	GO:0006915	apoptotic process
	GO:0012501	programmed cell death
	GO:0043066	negative regulation of apoptotic process
	GO:0043154	negative regulation of cysteine-type endopeptidase activity involved in apoptosis
	GO:2001237	negative regulation of extrinsic apoptotic signaling pathway
	GO:1902041	regulation of extrinsic apoptotic signaling pathway via DDR
	GO:0097296	activation of cysteine-type endopeptidase activity involved in apoptosis
	GO:0097194	execution phase of apoptosis
	GO:0010939	regulation of necrotic cell death
	GO:0051092	positive regulation of NF-kappaB transcription factor activity
	GO:0007519	skeletal muscle tissue development
	GO:0014842	regulation of skeletal muscle satellite cell proliferation
	GO:0043403	skeletal muscle tissue regeneration
MCL1	GO:1903378	positive regulation of oxidative stress-induced neuron apoptosis
	GO:0097192	extrinsic apoptotic signaling pathway in absence of ligand
	GO:0034097	response to cytokine
	GO:0007275	multicellular organism development
	GO:0019725	cellular homeostasis
PROK2	GO:0043066	negative regulation of apoptotic process
	GO:0001525	angiogenesis
	GO:0045765	regulation of angiogenesis
	GO:0007186	G-protein coupled receptor signaling pathway
	GO:0007218	neuropeptide signaling pathway
	GO:0006954	inflammatory response
	GO:0006935	chemotaxis
PRDX5	GO:0006915	apoptotic process
	GO:0043066	negative regulation of apoptotic process
	GO:0034614	cellular response to reactive oxygen species
	GO:0051354	negative regulation of oxidoreductase activity
	GO:0070995	NADPH oxidation
JTB	GO:0008637	apoptotic mitochondrial changes

$ Data from the Gene Ontology (2009).

Table S4. Gene Ontology (GO) terms for apoptosis-related biological processes that are over-represented in the list of significantly changed pro-apoptotic genes in the Table 5 in CD34+ cells from untreated GHD patients prior to GH-TS compared to their controls.

Human Gene	GO ID $	Qualified GO Term
FNTA	GO:0012501	programmed cell death
	GO:0006921	cellular component disassembly involved in execution phase of apoptosis
	GO:0007528	neuromuscular junction development
NFKBIZ	GO:0006351	transcription, DNA-templated
GADD45B	GO:0006915	apoptotic process
	GO:0006950	response to stress
	GO:0006469	negative regulation of protein kinase activity
	GO:0000186	activation of MAPKK activity
	GO:0000185	activation of MAPKKK activity
	GO:1900745	positive regulation of p38MAPK cascade
	GO:0046330	positive regulation of JNK cascade
	GO:0051726	regulation of cell cycle
	GO:0007275	multicellular organism development
TNF	GO:0043068	positive regulation of programmed cell death
	GO:0071550	death-inducing signaling complex assembly
	GO:0008625	extrinsic apoptotic signaling pathway via death domain receptors
	GO:0008630	intrinsic apoptotic signaling pathway in response to DNA damage
	GO:0006919	activation of cysteine-type endopeptidase activity involved in apoptotic process
	GO:0006927	transformed cell apoptotic process

Table S4. *Cont.*

Human Gene	GO ID $	Qualified GO Term
TNFRSF10C	GO:0008625	extrinsic apoptotic signaling pathway via death domain receptors
	GO:0033209	tumor necrosis factor-mediated signaling pathway
	GO:0007275	multicellular organism development
	GO:0032496	response to lipopolysaccharide
	GO:0007165	signal transduction
TNFRSF1A	GO:0008625	extrinsic apoptotic signaling pathway via death domain receptors
	GO:0008630	intrinsic apoptotic signaling pathway in response to DNA damage
	GO:0071550	death-inducing signaling complex assembly
	GO:0006954	inflammatory response
	GO:0050728	regulation of inflammatory response
	GO:0007165	signal transduction
	GO:0007249	I-kappaB kinase/NF-kappaB signaling
	GO:0007166	cell surface receptor signaling pathway
	GO:0019221	cytokine-mediated signaling pathway
	GO:0042127	regulation of cell proliferation
	GO:0007275	multicellular organism development
LITAF	GO:0006351	transcription, DNA-templated
	GO:0007165	signal transduction
	GO:0007568	aging
	GO:0042347	negative regulation of NF-kappaB import into nucleus
	GO:0043123	positive regulation of I-kappaB kinase/NF-kappaB signaling
CASP4	GO:0097193	intrinsic apoptotic signaling pathway
	GO:0070059	intrinsic apoptotic signaling pathway in response to RE stress
	GO:0050727	regulation of inflammatory response
	GO:1904646	cellular response to beta-amyloid
	GO:0006508	proteolysis
CASP8	GO:0006915	apoptotic process
	GO:0006919	activation of cysteine-type endopeptidase activity involved in apoptotic process
	GO:0012501	programmed cell death
	GO:0006921	cellular component disassembly involved in execution phase of apoptosis
	GO:0097191	extrinsic apoptotic signaling pathway
	GO:0097193	intrinsic apoptotic signaling pathway
	GO:0043154	negative regulation of cysteine-type endopeptidase activity involved in apoptotic process
	GO:0097296	activation of cysteine-type endopeptidase activity involved in apoptotic signaling pathway
	GO:1902041	regulation of extrinsic apoptotic signaling pathway via death domain receptors
	GO:1902042	negative regulation of extrinsic apoptotic pathway via DDR
	GO:0036462	TRAIL-activated apoptotic signaling pathway
	GO:0039650	suppression by virus of host cysteine-type endopeptidase activity involved in apoptotic process
	GO:0033209	tumor necrosis factor-mediated signaling pathway
	GO:0010803	regulation of tumor necrosis factor-mediated signaling pathway
	GO:0010939	regulation of necrotic cell death
	GO:0071550	death-inducing signaling complex assembly
	GO:0006508	proteolysis
	GO:0045862	positive regulation of proteolysis
	GO:0051603	proteolysis involved in cellular protein catabolic process
	GO:0043124	negative regulation of I-kappaB kinase/NF-kappaB signaling
	GO:0045651	positive regulation of macrophage differentiation
	GO:0030225	macrophage differentiation
	GO:0042113	B cell activation
	GO:0009409	response to cold
	GO:0071260	cellular response to mechanical stimulus
	GO:0071407	cellular response to organic cyclic compound
TNFAIP6	GO:0007165	signal transduction
	GO:0007267	cell-cell signaling
SMNDC1	GO:0006915	induction of programmed cell death by apoptosis

$ Data from the Gene Ontology (2009).
